# Characterizing and distinguishing the earliest woody euphyllophytes based on secondary xylem anatomy: method development and application

**DOI:** 10.1093/aob/mcaf122

**Published:** 2025-06-13

**Authors:** Emma Casselman, Alexandru M F Tomescu

**Affiliations:** Department of Biological Sciences, California State Polytechnic University – Humboldt, Arcata, CA 95521, USA; Department of Biological Sciences, California State Polytechnic University – Humboldt, Arcata, CA 95521, USA

**Keywords:** Secondary xylem, wood anatomy, fossil, Devonian, euphyllophyte, tracheid, gymnosperm, taxonomy, metric, quantitative comparison

## Abstract

**Background and Aims:**

The oldest vascular cambial growth (woody growth) has been recognized in several Early Devonian (ca. 410–395 Ma) euphyllophytes. Incomplete preservation of these fossils, in multiple cases, makes it difficult to evaluate their taxonomic diversity, in the absence of quantitative methods for distinguishing plants based on secondary xylem anatomy. In turn, this hinders understanding of their implications for the evolution of plant development. To develop and apply metrics that quantify secondary xylem anatomy and allow for conclusive comparisons, we investigated extant and Early Devonian fossil woody taxa.

**Methods:**

We developed multiple metrics for quantifying tracheid size as a function of position in the secondary xylem and tested them against a dataset of extant gymnosperm wood. The four metrics that showed consistent trends within taxa and captured differences among taxa were then applied to previously recognized Early Devonian fossil woody taxa and to previously undescribed Early Devonian woody specimens to compare them and evaluate taxonomic diversity and placement of the new fossils.

**Key Results:**

The four new secondary xylem metrics, considered alongside other anatomical characters, differentiated the previously recognized Early Devonian taxa from each other, allowed assignment of two of the new fossils to the previously described species *Franhueberia gerriennei* and separated two new woody euphyllophyte taxa.

**Conclusions:**

The metrics developed here for quantifying secondary xylem anatomy are effective in demonstrating conspecificity and in separating distinct woody taxa, especially in combination with data on the primary xylem and xylem rays. They provide a new method for assessing the taxonomic placement of fossils with incomplete preservation, opening up a new avenue for exploring fossil plant diversity and for characterizing anatomy, with implications for the evolution of plant development.

## INTRODUCTION

The origin and early evolution of woody growth are incompletely explored in the fossil record and thus are poorly understood. The Early Devonian record of woody growth, considered until not too long ago to be non-existent, is still sparse. Therefore, new specimens of woody plants from the Early Devonian can provide valuable new information on the origin and evolution of woody growth.

The Early Devonian witnessed one of the most extensive episodes of diversification among land plants (Cascales-Miñana *et al.*, 2010; Knoll, 2011; Cascales-Miñana, 2016). During this period, different plant lineages arising from the initial plexus of early tracheophytes with simple morphologies evolved structurally complex anatomy and body plans, including the evolution of stem, root, leaf organography (Matsunaga and Tomescu, 2017), differentiated histology and complex organization of vascular tissues (e.g. Gensel, 1984, 2022; Bickner and Tomescu, 2019; Toledo *et al.*, 2021), including tissues produced by secondary growth (Gerrienne *et al.*, 2011; Hoffman and Tomescu, 2013; Strullu-Derrien *et al.*, 2014). All of these represent adaptations that allowed vascular plants to further diversify into the floras that led to the rich diversity of modern vegetation (Kenrick and Crane, 1997).

Secondary growth from a vascular cambium, also known as woody growth, is identified in fossils based on a set of anatomical criteria that include: consecutive tracheids and interspersed parenchyma cells (xylem or wood rays) organized in radial files (secondary xylem, as seen in cross-sections); anticlinal divisions of cambial initials (multiplicative divisions) that led to the addition of new files of tracheids and wood rays; and a general organization of the secondary tissues into a longitudinal (axial) component and a radial component (e.g. rays) (Hoffman and Tomescu, 2013). Application of these criteria has led to the identification of vascular cambial growth in multiple plant lineages, recognized in rocks as old as the Middle Devonian or younger: lycopsids, cladoxylopsids, sphenophytes, rhacophytaleans, zygopterids, progymnosperms, stenokolealeans and seed plants (Andrews *et al.*, 1971; Cichan and Taylor, 1982, 1990; Toledo *et al.*, 2021).

Based on the diversity and timing of origin of these different lineages exhibiting woody growth, multiple independent origins of this developmental feature have been hypothesized (e.g. Cichan and Taylor, 1982; Spicer and Groover, 2010). This traditional perspective on the evolutionary timing of vascular cambial growth has undergone re-evaluation with the expansion of the fossil record of woody growth deeper in geological time, into the Early Devonian (Gerrienne *et al.*, 2011; Hoffman and Tomescu, 2013; Gensel, 2018). The discovery of Early Devonian euphyllophytes exhibiting woody growth much closer to the very origin of the euphyllophyte clade than the traditionally recognized lineages of the Middle Devonian led Hoffman and Tomescu (2013) to propose a single common origin of vascular cambial growth in the clade.

The Early Devonian fossil discoveries that led to these ideas include ten plant types with woody growth. *Armoricaphyton chateaupannense* (late Pragian, c. 408 Ma; Gerrienne et al., 2011; Strullu-Derrien *et al.*, 2014; Gerrienne and Gensel, 2016) from the Chalonnes Formation in France is the oldest fossil exhibiting woody growth. All other Early Devonian plants with woody growth are known from eastern Canada. Some of these were discovered in the Battery Point Formation (middle to late Emsian, c. 402–394 Ma) in Quebec: *Franhueberia gerriennei* (Hoffman and Tomescu, 2013), *Gmujij tetraxylopteroides* (Pfeiler and Tomescu, 2021) and *Kenrickia bivena* (Toledo *et al.*, 2021). Three other plants with secondary growth were described from the same unit: *Tainioxyla quebecana* (Bickner and Tomescu, 2019), which has been hypothesized to exhibit early stages of vascular cambial growth; a plant provisionally referred to as *Perplexa praestigians* (Pfeiler and Tomescu, 2023); and a plant referred to as a ‘*Psilophyton*-type axes’ by Gensel (2018). Finally, two other eastern Canadian fossil specimens with woody growth were reported from the early Emsian (c. 407–402 Ma) Campbellton Formation in New Brunswick by Gensel (2018); they are described as ‘New Brunswick plant A’ (first reported by Gerrienne *et al.*, 2011) and ‘New Brunswick plant B’ (Gensel, 2018).

Many of these taxa and plant types share terete centrarch protosteles. In *Armoricaphyton* the secondary xylem with multiplicative divisions possesses a radial tissue system consisting of two different types of rays (detailed below). Specimens similar to *Armoricaphyton* have been described as *Psilophyton*-type axes by Gensel (2018); these also show multiplicative divisions and may possess rays in the secondary xylem, although their preservation precluded ascertaining this feature. *Franhueberia* displays multiplicative divisions in the secondary xylem, as well as numerous uniseriate parenchymatous rays similar to those seen in most extant conifers (Hoffman and Tomescu, 2013), referred to hereafter as typical rays. Finally, ‘New Brunswick plant B’ has incompletely preserved central tissues that may represent a terete centrarch protostele and possesses a large amount of secondary xylem with multiplicative divisions and probable rays.

Two of the plants have four-lobed mesarch actinosteles. In *Gmuji* the actinostele probably possessed a central protoxylem strand. The secondary xylem of this plant shows multiplicative divisions and has a radial system best interpreted as consisting of parenchyma cells that replace several successive tracheids along the radial tracheid files (Pfeiler and Tomescu, 2021). This organization (hereafter referred to as *Gmujij*-type rays) is similar to some of the atypical rays seen in *Armoricaphyton* (Strullu-Derrien *et al.*, 2014) and has been proposed to result from the activity of a reversible cell fate switch that oscillates between parenchyma and sclerenchyma (tracheid) cell fate during differentiation of the derivatives of a cambial initial that form a radial cell file in the secondary xylem (Pfeiler and Tomescu, 2023). *Kenrickia* is a larger plant with a deeply lobed cruciform mesarch actinostele and a central protoxylem strand; its known secondary xylem forms a thin layer around the primary xylem (Toledo *et al.*, 2021).

Two other plants have elongated protosteles (as seen in cross-section). ‘New Brunswick plant A’ (Gensel, 2018) possesses multiplicative divisions and typical rays; the protoxylem, probably consisting of multiple strands, forms a line at the centre of the primary xylem. *Tainioxyla* (Bickner and Tomescu, 2019) is similar to ‘New Brunswick plant A’ in its elongated primary xylem, but the mesarch protoxylem strands that form a line at the centre of the primary xylem are discrete; the putative secondary xylem is barely developed, so its features (rays, multiplicative divisions) cannot be ascertained. Lastly, *Perplexa* (Pfeiler and Tomescu, 2023) has a terete protostele with multiple mesarch protoxylem strands toward its periphery and secondary xylem with multiplicative divisions but is lacking typical rays. Instead, in *Perplexa* the radial component of the secondary xylem is represented by radially oriented or radially widened tracheids and radially curved tracheid tips.

Lastly, fossils discovered in the Battery Point Formation and assigned by Banks *et al.* (1975) to *Psilophyton dawsonii* have not been interpreted to have secondary growth; they show possible evidence of multiplicative divisions but seemingly lack rays.

All the plants enumerated above possess *Psilophyton*-type (also known as P-type) tracheids – which have been described as scalariform bordered pitting with multiperforate apertures by Gensel (2018) – that indicate euphyllophyte affinities. The sole possible exception is ‘New Brunswick plant B’, in which the patterns of secondary wall thickening have not been documented.

A number of newly identified specimens in the Battery Point Formation of Quebec (Canada) stand out by their anatomical features (extent of secondary growth, tracheid size) from previously recognized woody taxa. This suggests that they could represent one or multiple new taxa, and clarifying this requires detailed anatomical description and comparisons among the new specimens and with other coeval woody taxa. Because of the specific features of these new fossils – lack of preservation of the primary xylem and extraxylary tissues, in most cases – such comparisons can only involve anatomical features of the secondary xylem. Furthermore, given the overall similarity of the secondary xylem of these early woody euphyllophytes, which consists of simple tracheids with P-type thickenings on all walls and fine uniseriate rays, conclusive comparisons that could identify differences between potentially distinct taxa require quantitative approaches. However, no quantitative approaches have been developed, to date, to achieve this. Consequently, this study has three components. The first involved analysis and comparisons of the wood of living taxa to develop and test the taxonomic discerning power of different secondary xylem metrics. This resulted in establishment of a method for conducting quantitative comparisons of wood anatomy, in order to assess similarity based on secondary xylem features. The second component involved testing the taxonomic discerning power of the newly developed method on previously described Early Devonian woody taxa. The third component consisted of applying the new method to the new fossil woody specimens from the Battery Point Formation to explore their relationships to each other and to the woody taxa already described from the Early Devonian. Specifically, the questions we addressed in this third step are: (1) how the new specimens compare to existing taxa and if they represent new species or new samples of existing species; and (2) the breadth of anatomical diversity encompassed by the woody taxa recognized to date in the Early Devonian. Answers to these questions have implications for our understanding of the evolution of secondary growth.

## MATERIALS AND METHODS

### Materials

**Fig. 1.**
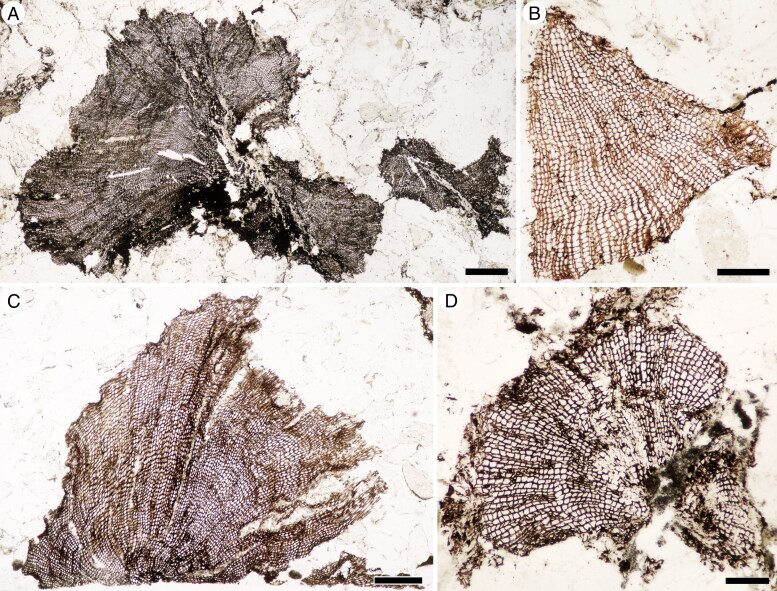
Woody plants from the Battery Point Formation. (A) Specimen 6; USMN 557840 Gtop 5b; this specimen has the second largest amount of secondary xylem of the six new specimens, but the central primary xylem is not preserved; note section of secondary xylem detached from the main axis (right). Scale bar = 500 μm. (B) Specimen 1; USNM 557840 Cbot 7a; only a wedge of secondary xylem with no primary xylem preserved. Scale bar = 300 μm. (C) Specimen 2; USNM 557839-2b Atop 845d; specimen with the largest amount of secondary xylem of the six new specimens; although is represents only half of the secondary xylem cylinder of an axis, this specimen preserves partially the central primary xylem (see Fig. 3A and 3B). Scale bar = 500 μm. (D) Specimen 4; USNM 557840 Fbot 8a; note partial preservation of tissue around the periphery of the secondary xylem. Scale bar = 200 μm.

**Fig. 2.**
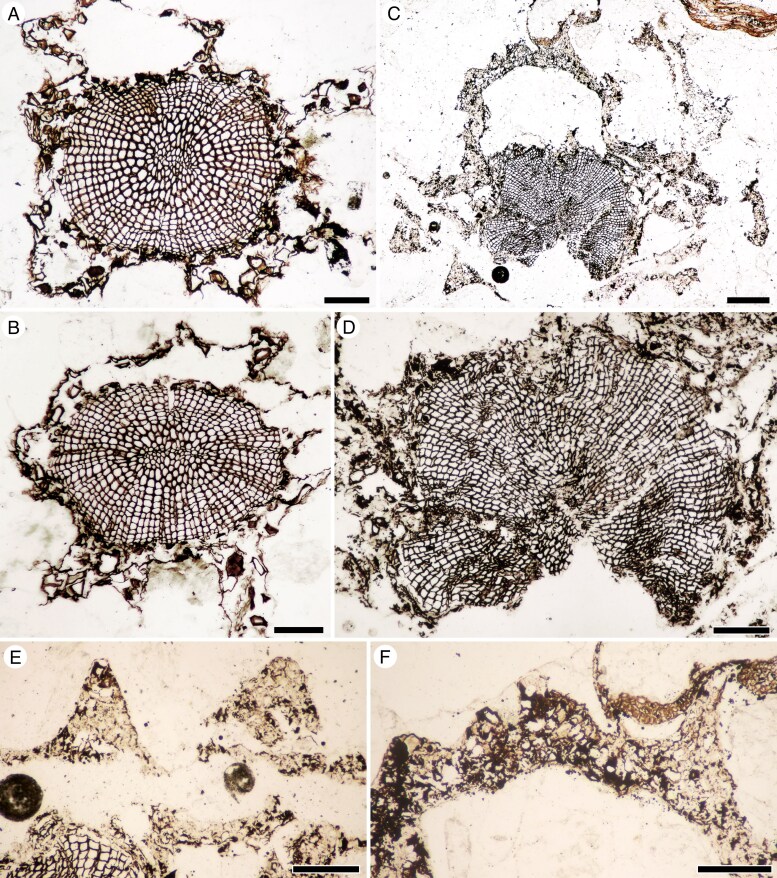
Woody plants from the Battery Point Formation. (A, B) Specimen 3; USMN 557839-2b Ctop 1a and USNM 557839-2b Bbot 1a, respectively; note well-preserved primary xylem with centrarch maturation (section in B basal to a branching point shows two protoxylem strands) and presence of extraxylary tissues including large sclerenchyma cells with thick secondary walls. Scale bars = 200 μm. (C, D) Specimen 5; USNM 557840 Htop 52b and USMN 557840 Htop 49b, respectively; note incompletely preserved central xylem and presence of extraxylary tissues, both immediately adjacent to secondary xylem (in D) and as a distorted layer of outer cortex at variable distance from the axis centre (in C). Scale bars = 350 μm (C), 200 μm (D). (E) Detail of (C). Two conical protrusions of the outer cortex; note presence of thick-walled cells in the protrusions and layer of tissue adjacent to the secondary xylem. Scale bar = 200 μm. (F) Specimen 5. Several protrusions of the outer cortex; note variable thickness of cell walls in the outer cortex and long, slender tip of the protrusion pointing to the top right corner of the image, which is in contact with part of an unrelated plant axis. USNM 557840 Htop 49b. Scale bar = 200 μm.

**Fig. 3.**
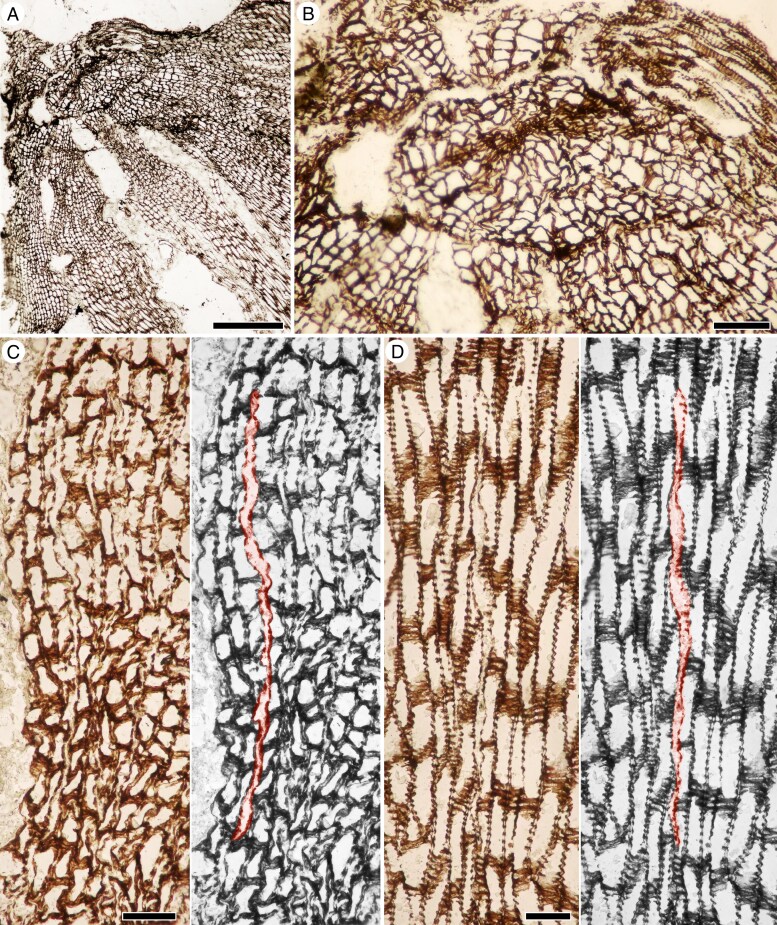
Primary xylem and rays of Specimen 2. (A) Small terete strand of primary xylem (top left) surrounded by incompletely preserved secondary xylem. USNM 557839-2b Atop 915d. Scale bar = 400 μm. (B) Detail of (A). Note centrarch maturation of the primary xylem. Scale bar = 100 μm. (C, D) Well-defined typical uniseriate rays. USNM 557839-2b Atop 915d. Scale bars = 50 μm.

**Fig. 4.**
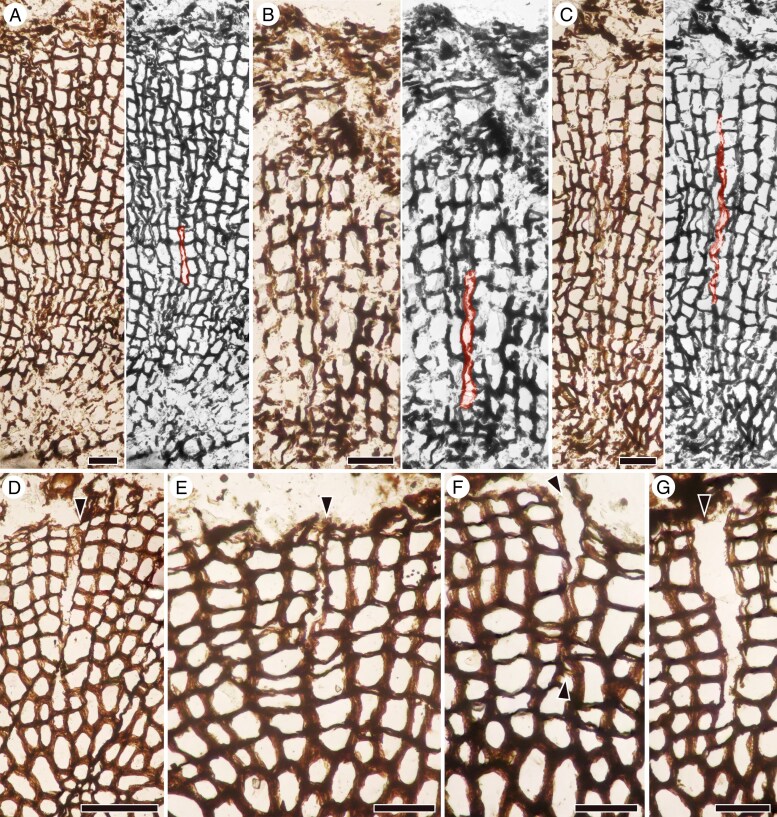
Rays of the new woody specimens. (A, B) Specimen 4; *Gmujij*-type (tracheid replacement) uniseriate rays. USNM 557840 Fbot 3a and USNM 557840 Fbot 4a, respectively. Scale bars = 50 μm. (C) Specimen 5; typical uniseriate ray. USNM 557840 Htop 27b. Scale bar = 50 μm. (D, E) Specimen 3; typical uniseriate rays. USMN 557839-2b Bbot 5a and USMN 557839-2b Bbot 9a, respectively. Scale bar = 100 μm (D), 50 μm (E). (F, G) Specimen 3; *Gmujij*-type rays (ray in F starting at the lower arrowhead is crushed in its lower half). USMN 557839-2b Bbot 22a. Scale bars = 50 μm.

**Fig. 5.**
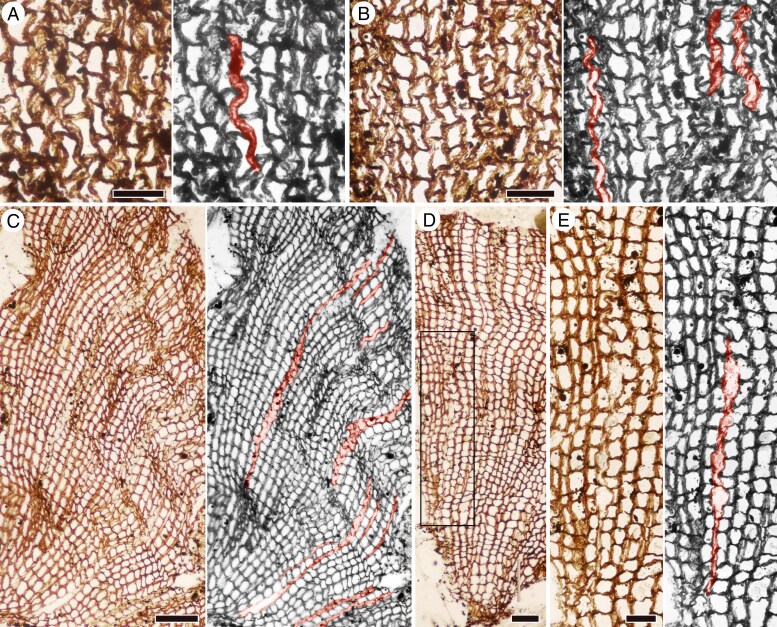
Typical uniseriate rays of the new woody specimens. (A, B) Specimen 6; USMN557840 Gtop 5b. Scale bars = 40 μm (A), 50 μm (B). (C–E) Specimen 1; (E) is detail of (D). USNM 557840 Dtop 5a (C) and USNM 557840 Cbot 7a (D, E). Scale bars = 150 μm (C), 100 μm (D), 50 μm (E).

The new fossil material studied consists of six fragments of plants with woody growth (Figs 1–5; Table 1) preserved by calcium carbonate permineralization in the fluvial sediments that form the Battery Point Formation. The specimens are preserved in ‘cobbles’ that are part of the collections of the U.S. National Museum of Natural History – Smithsonian Institution. The rocks were collected in the 1960s by the late Dr Francis Hueber from the south shore of Gaspé Bay (Quebec, Canada), in strata dated to the middle to late Emsian (Hoffman and Tomescu, 2013). Specimen collection numbers are as follows: Specimen 1, USNM 557840 Cbot a and Dtop a; Specimen 2, USNM 557839-2b Atop d; Specimen 3, USMN 557839-2b Bbot a and Ctop a; Specimen 4, USNM 557840 Fbot a; Specimen 5, USNM 557840 Htop b; and Specimen 6, USNM 557840 Fbot c, Gtop b (G1top), Gbot (G1bot) and Htop e.

**Table 1.** Comparison of new Battery Point Formation (BPF) specimens (1–6) and previously described Early Devonian fossil taxa. P-type refers to *Psilophyton*-type tracheid pitting.

Taxon	Primary xylem	Tracheid pitting	Maximum radial tracheid size (µm)	Maximum tangential tracheid size (µm)	Rays	Secondary xylem thickness (mm)	Secondary xylem thickness (max. tracheids in a file)	Multiplicative divisions
*Armoricaphyton chateaupannense*	Centrarch haplostele	P-type	100	50	Diverse	0.45	15	Present
*Franhueberia gerriennei*	Centrarch haplostele	P-type	41	29	Uniseriate	0.70	25	Present
*Psilophyton dawsonii*	Centrarch haplostele	P-type	100	125	Absent	0.45	10	Absent
*Gmujij tetraxylopteroides*	Mesarch actinostele	P-type	48	32	Uniseriate	0.32	13	Rare
BPF Specimen 1	?Centrarch haplostele	P-type	47	50	Typical, uniseriate	1.54	>41	Present
BPF Specimen 2	Centrarch haplostele	P-type	50	30	Typical, uniseriate	3.66	>95	Present
BPF Specimen 3	Centrarch haplostele	P-type	40	45	Typical, uniseriate + *Gmujij*-type	0.47	12	Present
BPF Specimen 4	?Centrarch haplostele	P-type	37	42	Rare, *Gmujij*-type	0.94	33	Present
BPF Specimen 5	?Centrarch haplostele	P-type	40	32	?Typical, uniseriate	0.72	21	Present
BPF Specimen 6	?Centrarch haplostele	P-type	42	37	Typical, uniseriate	2.48	>81	Present

Previously described fossil taxa included in comparisons are: *Gmujij tetraxylopteroides* (Pfeiler and Tomescu, 2021), *Franhueberia gerriennei* (Hoffman and Tomescu, 2013), *Armoricaphyton chateaupannense* (Strullu-Derrien *et al*., 2014) and fossils assigned by Banks *et al*. (1975) to *Psilophyton dawsonii*. We have selected these species for comparisons because (1) they are the oldest formally described plants with secondary growth (or radially aligned tracheids) – three of them reported from the same rock unit as our specimens (*Franhueberia*, *Gmujij* and *Psilophyton dawsonii*; Battery Point Formation) and one a few million years older than those (*Armoricaphyton*); and (2) because these plants are the most similar, among Early Devonian woody plants, to our new specimens in overall size, type of preservation and tracheid type (*Psilophyton*-type tracheids); other Early Devonian woody plants were not included in the study because they can be easily distinguished from our specimens based on their primary xylem architecture (*Tainioxyla*, *Kenrickia*) or their particular secondary xylem anatomy (*Perplexa*). Measurements of tracheids of these taxa were performed on published images (and on images of *Armoricaphyton* provided by the late Dr Philippe Gerienne).

Living taxa included in comparisons to develop and test the taxonomic discerning power of different metrics are *Ginkgo*, *Pinus*, *Sequoia* and *Ephedra*. We selected these four taxa because they sample a wide range of living gymnosperm taxonomic diversity and the secondary xylem of the earliest woody euphyllophytes is similar to that of living gymnosperms in consisting only of tracheids (despite the presence of vessel elements in the wood of *Ephedra*, in early developmental stages the secondary xylem differentiates exclusively as tracheids and is therefore comparable to the wood of the other gymnosperms). For *Ginkgo* and *Pinus* we measured secondary xylem of both stem and root and for *Sequoia* and *Ephedra* we measured only the wood of stems. For the stem or root of each of the four genera we measured multiple radial files from only one specimen, in slides available in the plant anatomy teaching collection at Cal Poly Humboldt. All the specimens measured were young stems or roots (<1 cm in diameter), and in each radial file we measured secondary xylem tracheids starting at the contact with the primary xylem.

### Fossil processing and observations

The new fossils were studied in sections made specifically for this study using the cellulose acetate peel technique (Joy *et al.*, 1956). Specimens for study under high-magnification light microscopy were mounted on slides using Eukitt (O. Kindler, Freiburg, Germany). The material was imaged using a Nikon Coolpix 8800VR digital camera mounted on a Nikon E400 compound microscope and an Olympus Dp73 digital camera mounted on an Olympus SZX16 microscope.

### Comparisons among taxa and specimens

In fossilized plants, xylem is one of the best preserved tissues, due to lignification of the cells’ secondary walls, which increases their resistance to decomposition. Thus, xylem is a major feature in descriptions and comparisons of Early Devonian plants (e.g. Pfeiler and Tomescu, 2021). Moreover, because of the thorough documentation of xylem (both primary and secondary) in extant and extinct lineages, xylem characters can be used for comparisons among previously described species and new specimens, extinct and extant.

Some of the specimens studied here preserve primary xylem and (rarely) extraxylary tissues. However, the preservation of these tissues is inconsistent among the different specimens and therefore unreliable for comparison. Because of that, our study relies heavily on comparisons of secondary xylem anatomy. Nevertheless, the comparisons between the new specimens and between them and previously described species employ both qualitative and quantitative characters.

The qualitative characters examined are:

- stele type (not observed in all specimens)
- protoxylem position (not observed in all specimens)
- metaxylem tracheid pitting (not observed in all specimens)
- secondary xylem tracheid pitting
- presence/absence of rays and ray type
- presence/absence of multiplicative divisions

The quantitative measurements are:

- xylem overall diameter (mm)
- primary xylem diameter (mm) (not measurable in all specimens due to incomplete preservation)
- metaxylem tracheid size – maximum size (µm) (not measurable in all the fossil specimens due to incomplete preservation)
- secondary xylem thickness – absolute (µm) and measured as maximum number of cells counted along a radial file of tracheids
- secondary xylem tracheid size – maximum size (µm), measured in radial and tangential directions
- ray size (µm) (not measured in all specimens)

### Quantifying tracheid size as a function of position in the secondary xylem for comparisons

Because plant cells, once produced in a given location by cell division, are usually immovable, the patterning of cells in the secondary xylem – especially in terms of their relative arrangement and size – records the history of cambial activity and thus can be used to characterize the dynamics of vascular cambial growth. In turn, because species are differentiated, among others, by their phenotypes, and because the latter are the result of development, characterizing development (such as by quantitative measures) and comparing it among species and individuals can inform taxonomy, which has implications for understanding patterns of diversity and, ultimately, evolution. Because of these reasons and because most specimens of early woody plants preserve little (if anything) other than secondary xylem, the bulk of our efforts focused on developing, testing and applying a method for quantitatively characterizing and comparing secondary xylem features that reflect the development of this tissue, for taxonomic purposes.

Our sampling approach for quantifying secondary xylem development was shaped by the objects and objectives of our study: comparing Early Devonian woody plants with different qualities of preservation to understand their taxonomic affinities. In this system, each individual specimen is treated as a potentially new taxon until proven differently, which limits the amount of data that can be collected. This limitation is compounded by preservational limitations: not all the specimens that have radially aligned tracheids and, therefore, potentially woody growth, are preserved equally well. In fact, our choice of specimens to include in the study had to address the question: What is the minimum size of a dataset of tracheid measurements for a specimen that allows for comparing its secondary xylem metrics using quantitative statistics? In the end, we had to address the trade-off between setting high standards for quality of preservation and the number of specimens that can be included in analyses. Indeed, some of the new specimens we identified initially in the Battery Point Formation for inclusion in the study turned out, upon closer inspection, to not have more than two or three well-preserved consecutive secondary xylem tracheids in more than one or two radial files; those specimens were not included.

In selecting the radial files for tracheid measurements, we took into account several considerations:

(1) Whenever available, we selected for measurement radial files at least ten tracheids long. However, in some instances, file length was limited (in fossils) by the availability of well-preserved tracheids.
(2) A limitation was imposed by multiplicative (anticlinal) divisions: in the majority of cases (especially in radial files >10 tracheids long), the highest-ranked tracheid included in measurements was the tracheid internal to the tracheid adjacent to the newly formed to files of tracheids. This was to exclude the effects that the growth of the initials of the two new files may have had on the tangential size of the tracheid immediately adjacent to them, which was still growing, prior to differentiating, at the same time as those initials.
(3) When possible, we avoided radial files immediately adjacent to files that had early multiplicative divisions. This was to avoid potential effects due to the transition from a single adjacent file to two adjacent tracheid files, on the tangential size of the measured file.
(4) For similar reasons as above, we avoided as much as possible measuring radial files immediately adjacent to ‘inserted’ tracheid files (i.e. files sectioned close to the tapered tips of their tracheids), as well as tracheid files immediately adjacent to rays.
(5) We avoided tracheid files that were much wider or much narrower – and, thus, not as representative of the overall anatomy and development of the plant – than the majority of the files in the specimen.
(6) Lastly, we avoided files that included obvious tracheids of anomalous size due to cell division or cell growth accidents; such files were recognized by the random presence of tracheids whose size and shape were inconsistent with those of tracheids in the same radial file and in immediately adjacent files.

Of the new specimens included in the study, Specimen 4 had the lowest number of measurable tracheid files (three files) with 4–10 tracheids each, for a total of 19 tracheids measured (Supplementary Data Sheet S1); as shown below, this dataset was too small to allow for conclusive comparisons with other specimens and taxa. Specimen 1, where we were able to measure 5–9 tracheids in each of five radial tracheid files for a total of 35 tracheids measured (Sheet S1), lended itself to conclusive comparisons, thus setting a putative lowest limit for the size of a useful dataset. The other four fossil specimens from the Battery Point Formation provided between eight and 14 tracheid files for measurements, with totals of 57–94 measured tracheids (Sheet S1). Similar to these numbers, in each of the four previously described Early Devonian taxa we were able to measure totals of 60–105 tracheids in ten files per taxon (with 4–18 tracheids per file) (Sheet S1). That such secondary xylem dataset sizes are sufficient for conclusive quantitative comparisons is demonstrated by their effectiveness in separating the four taxa, by themselves or in combination with other anatomical data.

To maintain sampling consistency with the fossil material, we aimed for measuring ten radial tracheid files in one specimen of each living taxon/organ, although in the *Pinus* stem we measured 12 tracheid files, whereas in the *Pinus* root and *Sequoia* stem we were only able to measure nine tracheid files that contained at least eight tracheids. This resulted in comparable sample sizes of 86–133 total tracheids measured per living taxon (Supplementary Data Sheet S1).

For proof of concept, we first applied multiple exploratory metrics to the wood of four living gymnosperms (*Ginkgo*, *Pinus*, *Sequoia* and *Ephedra*). Subsequently, we applied those metrics demonstrated to be effective in separating the living taxa, to the dataset of fossil taxa – both to previously described species, as a way to test the results obtained in the proof of concept phase involving living taxa, and to the new Battery Point Formation specimens. Among the new early woody euphyllophyte specimens, one (Specimen 3) is conspicuously different from the others under visual examination, so distinguishing it from them would not require statistical comparisons of quantitative data on its secondary xylem; this specimen was included in the analysis as another way of testing the discerning power of the metrics developed here.

To characterize cambial growth dynamics in our new specimens and identify effective means of differentiating taxa based on quantitative features of secondary xylem anatomy and development, we used different metrics of tracheid size as a function of position. Measurements were performed using ImageJ (US National Institutes of Health; https://imagej.net/ij) on digital images prepared in Adobe Photoshop (San Jose, CA, USA). Calculations, analyses and construction of graphs were performed using Microsoft Excel and NCSS 2024 statistical software (NCSS, LLC, Kaysville, UT, USA; ncss.com/software/ncss).

**Fig. 6.**
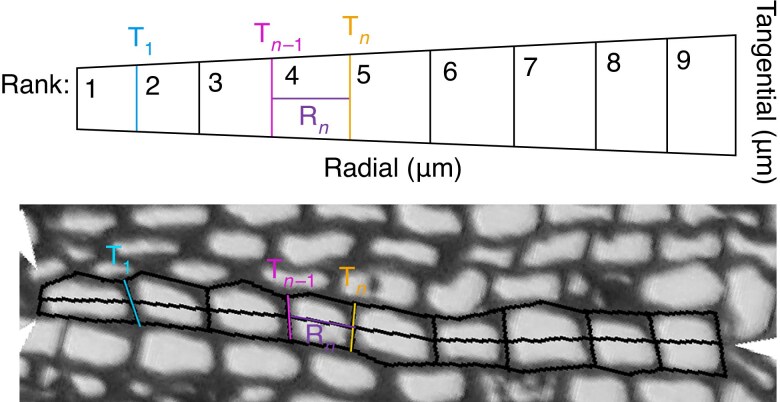
Measurements of variables used to characterize tracheid size as a function of position. The tangential (T*_n_*) size of tracheid of rank *n* is measured on its outer periclinal wall.

The two primary variables measured for each secondary xylem tracheid are tangential (T) and radial (R) size. The tangential size of a tracheid was measured along its outer periclinal wall because it provided for more consistency, given that (1) in many cases the shape of tracheid lumens and therefore tangential sizes measured elsewhere other than strictly along their periclinal walls is more strongly influenced by the shape of tangentially adjacent tracheids; and (2) especially in the fossil material tracheid lumens can be distorted, sometimes heavily, whereas their tangential walls typically maintain their integrity and size. The radial size of tracheids was measured along their radial midline. The position of each tracheid was recorded as its rank (*n*) in a radial file, starting with the secondary xylem tracheid immediately adjacent to the primary xylem, i.e. the first tracheid in the file, in developmental sequence, and going outwards (Fig. 6).

When the scarcity of radial files with well-preserved tracheids required measurement of files that included distorted tracheids, the measurements of the latter were corrected based on comparisons with radially adjacent tracheids, using the proportionality between the length of their radial walls and their measured radial size. For example, reconstructing the radial size (R*_n_*) of the distorted tracheid *n* in Supplementary Data Fig. S1 involved the following calculations:


(1) $$\frac{a_{n+1}+b_{n+1}}{R_{i}}=RP_{n+1}^{a,b}$$

where *a_n_*_+1_ + *b_n_*_+1_ is the total length of the radial wall of undistorted tracheid *n* + 1 measured on one of its sides and $RP_{n+1}^{a,b}$ is the ratio between the total length of the radial wall and the measured radial size of tracheid *n* + 1, based on measurement of the *a* + *b* side.

The accuracy of *RP* can be improved by calculating it for one or two more tracheids, *n* + 2 and *n + 3*. An average $\bar{RP}$ can then be calculated by adding the $RP^{a,b}$ for all the tracheids and dividing the sum by the number of tracheids.


(2) $$\bar{RP}=\frac{\sum_{i=n+1}^{n+x}\;RP_{i}^{a,b}}{x}\;\mathrm{where}\;x=2\;\mathrm{or}\;3,\mathrm{typically}$$

The average $\bar{RP}$ is then applied to tracheid *n* to reconstruct its undistorted radial size, based on measurements of its radial wall portions measured on the same side $R_{n}^{a,b}$.


(3) $$R_{n}^{a,b}=\frac{a_{n}+b_{n}}{{\bar{RP}}^{a,b}}$$

Calculations (Eqns 1–3) are then repeated using the opposite side of the tracheid file (*c* + *d*). Finally, the reconstructed R*_n_* is calculated as an average of the two values (Eqn 4):


(4) $$R_{n}=\frac{R_{n}^{a,b}+R_{n}^{c,d}}{2}$$

To characterize tracheid size as a function of position (rank, denoted as *n*) we used the two primary variables, T and R, as well as variables derived from the latter (Fig. 6).

1) The tangential size of tracheid of rank *n* (T*_n_*) relative to that of the first tracheid in the file (T_1_)
  (5) $$T_{n}\;/\;T_{1}$$
2) The tangential size of tracheid of rank *n* (T*_n_*) relative to that of the preceding tracheid in the file (T*_n_*_−1_)
  (6) $$T_{n}\;/\;T_{n-1}\;$$
3) The tangential size of tracheid of rank *n* (T*_n_*) relative to its radial size (R*_n_*)
  (7) $$T_{n}\;/\;R_{n}$$
4) The relative increase in tangential size (RIT*_n_*): the increase in tangential size of tracheid of rank *n* (between its outer and inner periclinal walls) per unit of radial size of that tracheid (Eqn 8):
  (8) $$RIT_{n}\;=\;\frac{T_{n}\;-\;T_{n-1}}{R_{n}}$$

This metric mitigates noise introduced by the variability of radial tracheid size – a measurement that is influenced by the environment. The best example of this influence is the difference in radial tracheid size between early and late wood cells in the same tracheid file.

5) The relative tangential increase in size of tracheid of rank *n* (RIT*_n_*) relative to that of the preceding tracheid in the file (RIT*_n_*_−1_):
  (9) $$\frac{RIT_{n}}{RIT_{n-1}}$$

These variables were represented as scatter plots – one for each tracheid file – and as box plots, where each box plot represents the values of the respective variable pooled together for each tracheid rank.

Additionally, we compared several variables via regressions:

6) The tangential size of tracheid of rank *n* (T*_n_*) plotted against the preceding tracheid in the file (T*_n_*_−1_)
7) To quantify tracheid size as a function of position relative to the primary xylem but free of rank, we plotted tangential tracheid size of tracheid of rank *n* (T*_n_*) against its distance from the primary xylem (or cumulative R: R_1_ + R_2_ + … + R*_n_*).
8) The relative tangential increase in size of tracheid of rank *n* (RIT*_n_*) plotted against the radial measurement of that tracheid (R*_n_*).
9) The relative tangential increase in size of tracheid of rank *n* (RIT*_n_*) plotted against the tangential measurement of that tracheid (T*_n_*).
10) We also calculated the mean increase in tangential size per tracheid in each file of tracheids (MI) by subtracting the tangential size of the first tracheid measured (T_1_) from that of the last tracheid in the file (T*_n_*) and dividing the result by the number of tracheids measured between and including the two. MI, which is a measure of the degree of centrifugal flare of a tracheid file and therefore reflects the increase in tangential size of the cambial initials as a function of distance from the primary xylem, is calculated for each tracheid file using the formula (Eqn 10):
  (10) $$MI=\frac{T_{n}-T_{1}}{n-1}$$

The MI values of each taxon are then pooled into a box plot representative of that taxon. These pooled MI values of individual taxa were compared statistically in terms both of their means, using the Aspin–Welch unequal-variance *t*-test, and of their distributions using the Kolmogorov–Smirnov test, in NCSS. Absence of a statistically significant difference was inferred in cases where the null hypothesis could not be rejected at *P* < 0.05 by at least one of the two tests.

11) We also tested the impact of the number of tracheids measured in a file (i.e. T_1-4_, T_1-7_, T_1-10_) and of the sampling of different tracheid sequences along the same file (i.e. T_1-4_, T_4-7_, T_7-10_), on T*_n_* vs. T*_n_*_−1_ and on T*_n_* vs. cumulative R.

It is important to note that some of the metrics used here have been used previously by Cichan (1985*a*) and D’Antonio (2023). These include comparison of tangential tracheid size to that of preceding tracheids in a file and the increase of tangential tracheid size as a function of radius. Additionally, D’Antonio (2023) used the Kolmorogov–Smirnov test for identifying wood anatomical features that are developmentally meaningful.

## RESULTS

The measurements for each taxon, extant and fossil, as well as the specific tracheid files on which measurements were performed are shown in the Supplementary Data Sheets S1 and S2. Regression equations for all taxa are listed in the Supplementary Note.

### Qualitative comparisons

The new woody plant specimens (Figs 1–5; Table 1) share a number of features: P-type tracheids, multiplicative divisions in the secondary xylem and, probably, small centrarch haplosteles and presence of a radial tissue system in the secondary xylem. Although the primary xylem is only preserved in two of the specimens, the patterning of secondary xylem tracheids in the four others that lack primary xylem preservation strongly suggests that the primary xylem has small dimensions and was probably a haplostele. Presence of rays is hard to ascertain or characterize in several of the specimens due to suboptimal preservation. Nevertheless, available data indicate that the new specimens differ in their radial systems. Some of the specimens have typical uniseriate rays, as seen in *Franhueberia* and many modern gymnosperms, while others have *Gmujij*-type rays produced by replacement of tracheids with parenchyma cells (as described by Pfeiler and Tomescu, 2021). This observation suggests that despite some aspects of similarity, the new specimens may represent more than one taxon. The relatively broad range of tracheid sizes – 37–50 μm radially and 32–50 μm tangentially – are also consistent with taxonomic diversity among the new woody plant specimens. Collectively, these tracheids reach the upper limit of tangential tracheid sizes for Early Devonian woody plants, but they are still considerably smaller than the upper limit of radial tracheid sizes (100 μm in *Armoricaphyton*; Table 1).

### Quantitative comparisons

#### Proof-of-concept: extant gymnosperm genera

The scatter plots representing individual tracheid files (Supplementary Data Figs S2–S6) show, expectedly, relatively high variability which makes it difficult and unreliable to identify trends in the data, if present. For this reason, to capture potential overall trends in the data, we also represented the same data using series of box plots that summarize the same metric for each cell rank in all the cell files (Figs S2–S6).

The ratio of tangential tracheid size (T*_n_*) relative to that of the first tracheid in the file (T_1_) (Supplementary Data Fig. S2) increases overall with cell rank in all the living taxa (*Pinus*, *Sequoia*, *Ginkgo* and *Ephedra*), in both stems and roots (where sampled, i.e. *Pinus* and *Ginkgo*). This is expected given the geometry of tracheid files where tangential tracheid size increases centrifugally. The ratio of tangential size between each tracheid (T*_n_*) and the one preceding it in the file (T*_n_*_−1_) (Fig. S3) shows an overall constant trend. This is consistent with a constant growth ratio between successive tracheids, also predicted by the geometry of tracheid files. The variability between successive ranks is due primarily to variability in tracheid radial size along cell files and is most marked in the lower cell ranks of *Ginkgo* and *Ephedra* stems, as well as in the *Pinus* root throughout the ranked series (Fig. S3). The ratio of tangential tracheid size (T*_n_*) relative to that of its radial size (R*_n_*) (Fig. S4) shows different trends between the taxa: the stem and root of *Pinus* both have constant ratios throughout the ranked series (Fig. S4), whereas in the stems of *Sequoia*, *Ephedra* and *Ginkgo* the ratio increases; the root of *Ginkgo* tends to have constant ratios, but with significant variability in the lower and higher cell ranks. The ratio of relative increase in tangential tracheid size (RIT*_n_*) relative to the radial size (R*_n_*) shows no consistent pattern (Fig. S5): decrease with tracheid rank in *Sequoia* or relatively constant ratio but with widely different amounts of variability between successive ranks in the other taxa. Like in the case of T*_n_*/T*_n_*_−1_ (Fig. S2) the variability between successive ranks present in some of the taxa reflects variability in tracheid radial size along cell files. When the relative increase in tangential tracheid size (RIT*_n_*) is compared between successive tracheids (RIT*_n_*/RIT*_n_*_−1_) the plots show markedly constant ratios (Fig. S6) that reflect the colinearity of radial walls along tracheid files; in these graphs the outliers correspond to kinks or other irregularities affecting the collinearity.

**Fig. 7.**
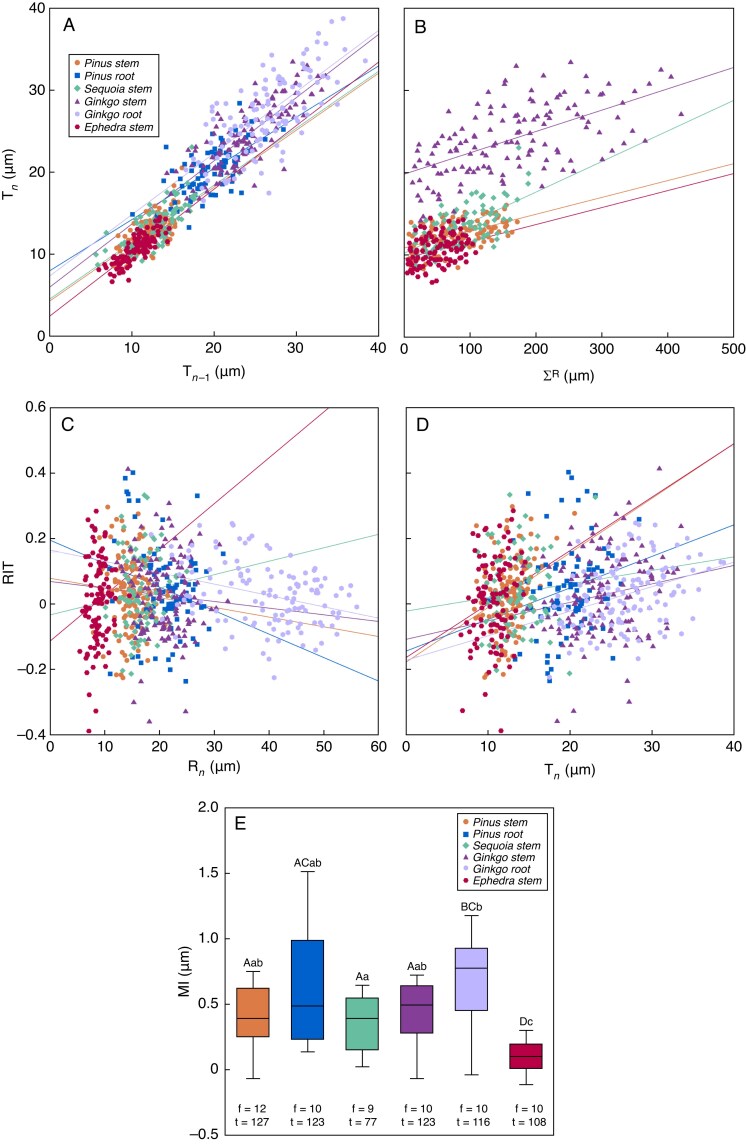
Extant taxa. Metrics considered for use in taxonomic comparisons. (A) The ratio of tangential size between each tracheid (T*_n_*) and the one preceding it (T*_n_*_−1_). (B) Tangential tracheid size (T*_n_*) against cumulative R. (C) The increase in tangential tracheid size (RIT*_n_*) relative to the radial size (R*_n_*). (D) The ratio of relative increase in tangential tracheid size (RIT*_n_*) against T*_n_*. (E) Mean increase in tangential tracheid size along radial files (MI). Letters indicate statistically significant differences between taxa (reject H_0_ at α = 0.05): uppercase letters = Aspin–Welch unequal-variance *t*-test; lowercase letters = Kolmogorov–Smirnov test for distributions. Regression lines in A–D were allowed to exceed the measured values for easier visual comparison of their position and slopes.

The tangential sizes of successive tracheids are relatively strongly correlated (T*_n_* vs. T*_n_*_−1_; Fig. 7A, Supplementary Note), especially when compared to the other correlations we explored.

However, there is considerable variation in the strength of these correlations (as reflected in their *R*^2^ values) between the strongest (*Pinus* root; *R*^2^ = 0.91) and the weakest correlation (*Ginkgo* root; *R*^2^ = 0.12). Nevertheless, except for the *Pinus* stem (*R*^2^ = 0.47), the remaining taxa have *R*^2^ > 0.60 (Supplementary Note). There is significant overlap between the clusters of data points of congeneric stems and roots (*Ginkgo*, *Pinus*), conspicuous partial separation of the *Pinus* root and *Ginkgo* clusters from each other and from those of the *Pinus*, *Sequoia* and *Ephedra* stems, which overlap to a great extent (Fig. 7A).

The regressions of tangential tracheid size (T*_n_*) against cumulative R for the stems of the four extant gymnosperm genera show relatively weak correlations (Supplementary Note). Nevertheless, they distinguish *Ginkgo* from the other three, whose distributions are broadly overlapping (Fig. 7B).

The relative increase in tangential tracheid size (RIT*_n_*) shows no correlation with the radial size of the tracheids (R*_n_*) (Fig. 7C). The taxa occupy roughly the same interval on the RIT*_n_* axis but show distinctive grouping by taxon along the R*_n_* axis. The *Ginkgo* root exhibits the widest variation and is separated from the other taxa, which are also partially separated as vertically spread clusters along this axis, toward the lower values; the most significant overlap is that between the *Sequoia* and *Pinus* stems (Fig. 7C).

A similar pattern of distribution, as well as lack of correlation, is seen in the relationship between the relative increase in tangential tracheid size (RIT*_n_*) and the tangential size of that tracheid (T*_n_*) (Fig. 7D).

The values of the mean increase in tangential size per tracheid (MI) vary widely in some taxa (notably the roots of *Ginkgo* and *Pinus*) and have the narrowest range in the *Ephedra* stem (Fig. 7E). They show significant overlap between some of the taxa (*Ginkgo*, *Pinus* and *Sequoia* stems), while distinguishing some of the taxa: *Ephedra* from all the other taxa; and *Ginkgo*, *Pinus*, and *Sequoia* stems from the roots of *Ginkgo* and *Pinus*. Interestingly, the stems and roots of the same genus overlap only partially. Overall, the MI values separate *Ephedra* from all the other taxa in terms of both mean and distribution. The other taxa are generally statistically similar to each other, with the exception of a statistical difference between *Sequoia* and *Ginkgo* root (different from one another in means and distributions) and a statistical difference between *Pinus* stem and *Ginkgo* root in terms of means.

We also explored how sampling along tracheid files affects the correlations and regressions for T*_n_* vs. T*_n_*_−1_ and for T*_n_* vs. cumulative R. For this, we compared subsets of the measurements along tracheid files with measurements of the entire files (tracheids 1–10), in the stems of the four genera (*Pinus*, *Sequoia*, *Ginkgo* and *Ephedra*). The subsets of measurements are:

- successive sets of measurements along tracheid files (i.e. T_1-4_, T_4-7_, T_7-10_) treated as independent samples and compared to T_1-10_ (Supplementary Data Fig. S7); and
- additive sets of measurements along tracheid files (i.e. T_1-4_, T_1-7_, T_1-10_) (Supplementary Data Fig. S8).

The regressions based on the successive sets of measurements are very similar overall (good overlap) to those obtained for the entire tracheid files, for both T*_n_* vs. T*_n_*_−1_ and for T*_n_* vs. cumulative R (Supplementary Data Fig. S7, Supplementary Note). The only exceptions for T*_n_* vs. T*_n_*_−1_ are *Pinus* T_4-7_ and T_7-10_ and *Sequoia* T_1-4_, which deviate slightly from the regressions based on the entire tracheid files (Fig. S7A and S7B, respectively). For T*_n_* vs. cumulative R, the exceptions are T_1-4_ in *Pinus* and especially *Ephedra*, and T_7-10_ in *Sequoia* (Fig. S7E, S7H and S7F, respectively).

In the case of regressions based on the additive sets of measurements, the subsets that deviate from the regressions obtained for the entire tracheid files are T_1-4_ in *Sequoia* and, markedly, T_1-7_ in *Ginkgo*, for T*_n_* vs. T*_n_*_−1_ (Supplementary Data Fig. S8B and S8C, respectively; Supplementary Note). For T*_n_* vs. cumulative R, deviations are present in T_1-4_ in *Pinus* and in *Ephedra* (Fig. S8E and S8H, respectively; Supplementary Note).

#### Proof-of-concept: methodological implications of analyses of extant gymnosperm genera

The proof-of-concept analyses of extant gymnosperms revealed that only some of the metrics applied describe trends in the data that are (1) internally consistent for individual taxa, with moderate ranges of variability, and (2) distinguish at least some of the taxa from the others. Specifically, when considered together, four metrics allow for differentiation amongst all the taxa. The four metrics are T*_n_* vs. T*_n_*_−1_, T*_n_* vs. cumulative R, RIT*_n_* vs. R*_n_*, and the mean increase in tangential size per tracheid (MI). T*_n_* vs. T*_n_*_−1_ separates the taxa into two groups, one consisting of *Pinus* root and *Ginkgo* stem and root, and the other of *Pinus*, *Sequoia* and *Ephedra* stems (Fig. 7A). T*_n_* vs. cumulative R definitively separates *Ginkgo* stem from the others and allows the *Sequoia* stem to be differentiated from *Pinus* (Fig. 7B). RIT*_n_* vs. R*_n_* shows separation of the taxa along the *x-*axis (Fig. 7C) and the MI plot separates *Ephedra*, on the one hand, and the roots, on the other, from the rest of the samples (Fig. 7E). Consequently, these metrics were selected for further application to the previously described Early Devonian fossils that have been demonstrated to represent distinct taxa, as a second proof-of-concept round.

#### Testing the method: previously described Early Devonian woody taxa

**Fig. 8.**
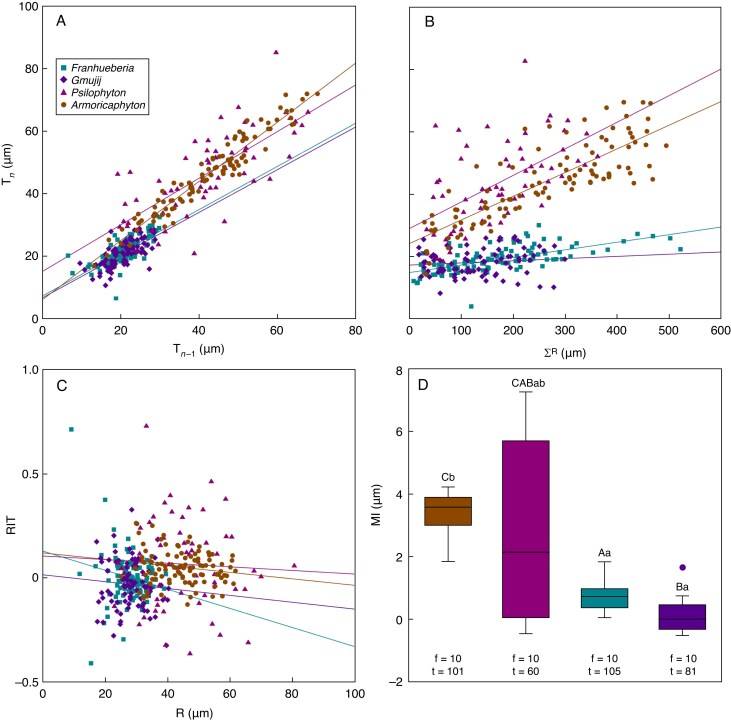
Comparisons of previously described fossil taxa. (A) The ratio of tangential size between each tracheid (T*_n_*) and the one preceding it (T*_n_*_−1_). (B) Tangential tracheid size (T*_n_*) against cumulative R. (C) The increase in tangential tracheid size (RIT*_n_*) relative to the radial size (R*_n_*). (D) Mean increase in tangential tracheid size along radial files (MI). Letters indicate statistically significant differences between taxa (reject H_0_ at α = 0.05): uppercase letters = Aspin–Welch unequal-variance *t*-test; lowercase letters = Kolmogorov–Smirnov test for distributions. Regression lines in A–C were allowed to exceed the measured values for easier visual comparison of their position and slopes.

The regressions of tangential tracheid size (T*_n_*) to the tangential size of the previous tracheid in the file (T*_n_*_−1_) show the same strong correlations for all four fossil taxa as seen in the extant taxa (Fig. 8A, Supplementary Note). *Psilophyton*, which is not thought to possess secondary growth and has the widest variability, overlaps considerably with *Armoricaphyton*. The two are distinct from *Gmujij* and *Franhueberia*, which overlap considerably with each other and are characterized by smaller and less variable tangential tracheid sizes.

The regressions of tangential tracheid size (T*_n_*) against cumulative R (Fig. 8B) show some overlap between *Armoricaphyton* and *Psilophyton* (which has the widest variability). Similar to the distinction seen in the T*_n_* vs. T*_n_*_−1_ regressions, *Armoricaphyton* and *Psilophyton* are clearly separated from *Gmujij* and *Franhueberia*, which overlap considerably with each other (*Franhueberia* exhibiting a wider variation in cumulative R).

The regressions of relative increase in tangential tracheid size (RIT*_n_*) against radial tracheid size (R*_n_*) (Fig. 8C) show separation along the R-axis between *Psilophyton* (showing the widest variability) and *Armoricaphyton*, on the one hand, and *Franhueberia* and *Gmujij* (tightly clustered together with lower overall values), on the other.

The mean increase in tangential tracheid size along radial files (MI) (Fig. 8D), as compared based on statistical tests of similarity, clearly separates *Armoricaphyton* from *Gmujij* and *Franhueberia*. The means of *Franhueberia*, *Gmujij* and *Armoricaphyton* are significantly different from each other, but none of them is statistically different from the mean of *Psilophyton*. In terms of MI distributions, *Franhueberia*, *Gmujij* and *Psilophyton* are not statistically different from each other, but all three are different from *Armoricaphyton*.

#### Testing the method: implications of analyses of previously described Early Devonian woody fossils

It is clear from the above comparisons that when used separately, none of the different metrics can unequivocally differentiate each of the four fossil taxa from the others. The different regressions show significant overlap between *Armoricaphyton* and *Psilophyton* and strong overlap between *Franhueberia* and *Gmujij*. The MI values differentiate only between some of the four taxa based on pairwise comparisons of the means and between others based on comparisons of their distributions.

However, when considered together, the four metrics differentiate each of the four taxa from the others reasonably well, as in the case of the living taxa. In cases where the distinctions are less marked, consideration of other anatomical data can lend additional support (Table 1). Thus, in the case of *Franhueberia* and *Gmujij*, which are statistically different from each other only in terms of their MI means, the primary xylem clearly separates the two taxa: it is terete in *Franhueberia* and four-lobed in *Gmujij*. The case of *Psilophyton* is also illustrative of the strength and weaknesses of metrics based on radially aligned tracheids. The clear separation of *Psilophyton* from *Franhueberia* and *Gmujij* based on the three types of regressions demonstrates the strength of the method. On the other hand, the regressions do not unequivocally separate *Psilophyton* from *Armoricaphyton* and neither do MI comparisons separate *Psilophyton* from any of the other three taxa. These data could be regarded as highlighting a weakness of the method tested here. However, the similarities between *Psilophyton* and the other three taxa that are implied by the different metrics are invalidated when considering the overall xylem anatomy of *Psilophyton*. For instance, the sampling of *Psilophyton* tracheids was limited to only a few tracheid files that were regular enough to be measured as equivalent to those of the other taxa. Even among these few regular files, the ranges of variation in all the metrics considered are much broader than those of the other taxa, although all the tracheid files considered are much shorter than those measured in the other taxa. Thus, the radially aligned xylem of *Psilophyton* encompasses much broader variability than that present in other taxa and that measured for these comparisons.

#### Applying the method to new fossil specimens

Specimen 4 provided only three radial tracheid files (totalling 19 tracheids) that were preserved well enough to allow for measurements. This is the smallest sample size of the six new specimens and affects the conclusiveness of comparisons between this specimen and specimens represented by larger samples. Thus, while Specimen 4 is included in the graphs, it is excluded from further discussions of the results.

**Fig. 9.**
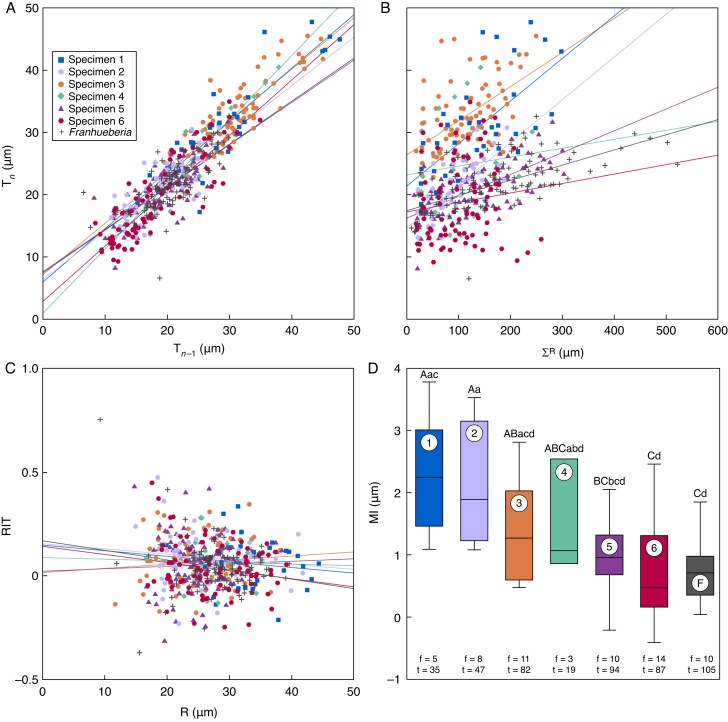
Comparisons between new woody specimens from the Battery Point Formation and *Franhueberia gerriennei*. (A) The ratio of tangential size between each tracheid (T*_n_*) and the one preceding it (T*_n_*_−1_). (B) Tangential tracheid size (T*_n_*) against cumulative R. (C) The increase in tangential tracheid size (RIT*_n_*) relative to the radial size (R*_n_*). (D) Mean increase in tangential tracheid size along radial files (MI). Letters indicate statistically significant differences between taxa (reject H_0_ at α = 0.05): uppercase letters = Aspin–Welch unequal-variance *t*-test; lowercase letters = Kolmogorov–Smirnov test for distributions. Regression lines in A–C were allowed to exceed the measured values for easier visual comparison of their position and slopes.

**Fig. 10.**
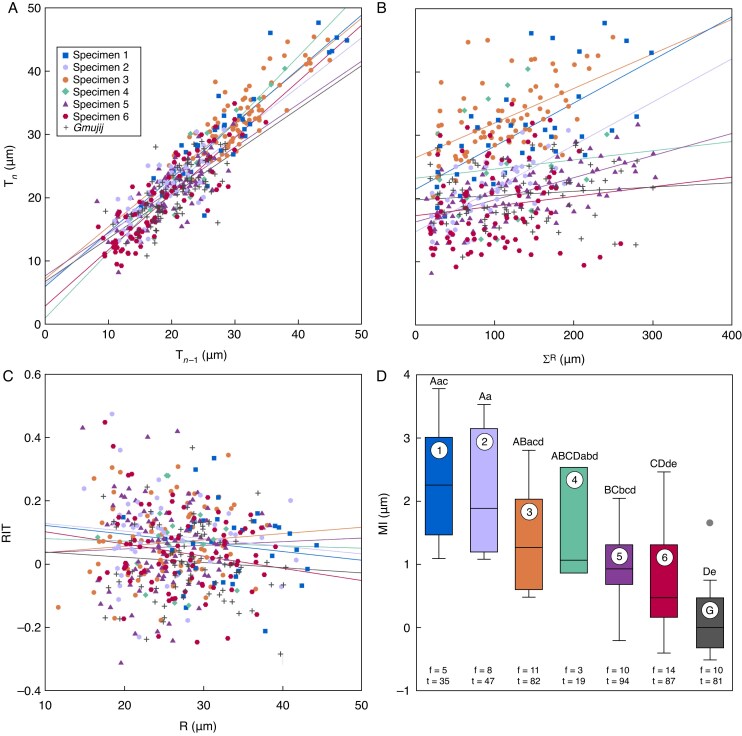
Comparisons between new woody specimens from the Battery Point Formation and *Gmujij tetraxylopteroides*. (A) The ratio of tangential size between each tracheid (T*_n_*) and the one preceding it (T*_n_*_−1_). (B) Tangential tracheid size (T*_n_*) against cumulative R. (C) The increase in tangential tracheid size (RIT*_n_*) relative to the radial size (R*_n_*). (D) Mean increase in tangential tracheid size along radial files (MI). Letters indicate statistically significant differences between taxa (reject H_0_ at α = 0.05): uppercase letters = Aspin–Welch unequal-variance *t*-test; lowercase letters = Kolmogorov–Smirnov test for distributions. Regression lines in A–C were allowed to exceed the measured values for easier visual comparison of their position and slopes.

**Fig. 11.**
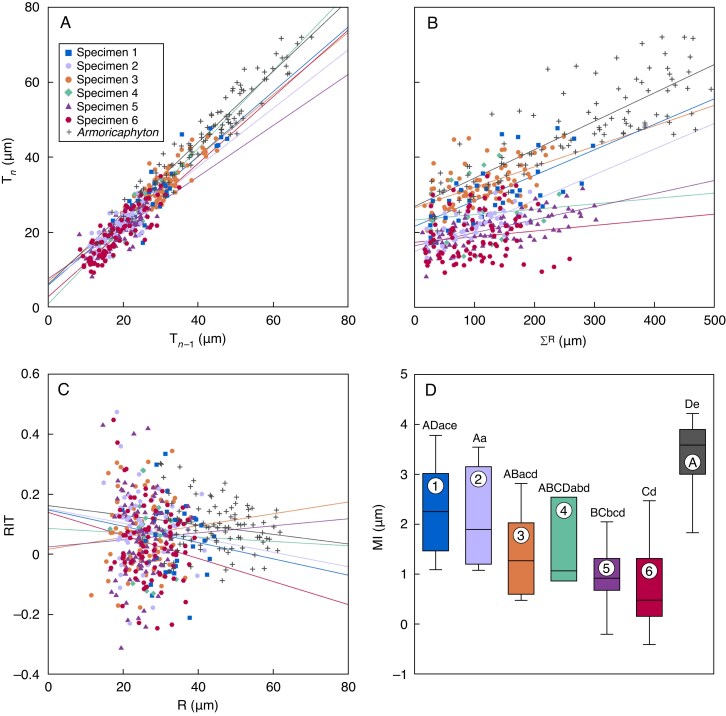
Comparisons between new woody specimens from the Battery Point Formation and *Armoricaphyton chateaupannense*. (A) The ratio of tangential size between each tracheid (T*_n_*) and the one preceding it (T*_n_*_−1_). (B) Tangential tracheid size (T*_n_*) against cumulative R. (C) The increase in tangential tracheid size (RIT*_n_*) relative to the radial size (R*_n_*). (D) Mean increase in tangential tracheid size along radial files (MI). Letters indicate statistically significant differences between taxa (reject H_0_ at α = 0.05): uppercase letters = Aspin–Welch unequal-variance *t*-test; lowercase letters = Kolmogorov–Smirnov test for distributions. Regression lines in A–C were allowed to exceed the measured values for easier visual comparison of their position and slopes.

The regressions of tangential tracheid size (T*_n_*) to the tangential size of the previous tracheid in the file (T*_n_*_−1_) show strong correlations for the six new fossil specimens (Supplementary Data Fig. S9A; Figs 9–11). The regressions of the six specimens overlap considerably, with only Specimen 5 standing out by its lowest slope.

The correlations between tangential tracheid size (T*_n_*) and cumulative R (Supplementary Data Fig. S9B; Figs 9–11) are not as strong as the T*_n_*/T*_n_*_−1_ correlations. The most obvious separation is between the three specimens with steeper regression slopes (Specimens 2, 3 and 1) and the two that have lower slopes (Specimens 6 and 5).

The correlations between the relative increase in tangential tracheid size (RIT*_n_*) and radial tracheid size (R*_n_*) (Supplementary Data Fig. S9C; Figs 9–11) are also weak. Whereas most specimens have relatively flat regression slopes, Specimen 6 stands out with a steeper negative slope.

The mean increase in tangential tracheid size along radial files (MI) (Supplementary Data Fig. S9D; Figs 9–11) separates Specimens 1 and 2 (with high values) from Specimen 6 (with the lowest values), based on sample variances (Aspin–Welch unequal-variance *t*-test). Specimen 3 is not statistically different from the high values group (Specimens 1 and 2), whereas Specimen 5 is not statistically different from Specimen 6. However, Specimens 3 and 5 are statistically similar to each other, thus forming a transitional group in terms of sample variances. The tests of similarity of sample distributions (Kolmogorov–Smirnov) only separate unequivocally Specimen 2 from Specimen 6, with the other specimens (1, 3 and 5) being statistically similar to either Specimen 1 or 6, and to each other.

### New fossil specimens compared to previously described fossil taxa

Of the five new specimens included in extensive comparisons (Specimen 4 was excluded, see above), Specimens 5 and 6 are statistically similar to *Franhueberia* by all our terms of comparison: variance and distribution of MI (Fig. 9D); T*_n_* vs. T*_n_*_−1_ regression (Fig. 9A), where Specimen 5 practically overlaps with *Franhueberia*; RIT vs. R regression (Fig. 9C), where Specimen 6 practically overlaps with *Franhueberia*; and the T*_n_* vs. cumulative R regression (Fig. 9B), where the regression line of *Franhueberia* bisects the angle formed by the regression lines of the two specimens. These results provide strong support for assignment of Specimens 5 and 6 to *Franhueberia*. Consistent with these comparative metrics, Specimens 5 and 6 are relatively similar to *Franhueberia* in maximum tracheid size and possess the same type of uniseriate rays (Table 1).

Specimen 3 is statistically similar to *Franhueberia* only in its MI data distribution (Fig. 9D), but it is among the most dissimilar from *Franhueberia* of the five specimens in terms of our other comparison metrics. However, Specimen 3 is also statistically similar to Specimen 5 (that has strongly supported *Franhueberia* identity) in its MI variance and distribution (Fig. 9D) and has the RIT vs. R regression closest to that of Specimen 5 (Fig. 9C). Thus, whereas some of our comparative results suggest that Specimen 3 could be a *Franhueberia* fragment, others suggest it is different from this genus. In support of the latter hypothesis, Specimen 3 has some of the largest tracheids in terms of tangential size (surpassed only by those of *Armoricaphyton*; Table 1), whereas *Franhueberia* has the smallest tracheids by tangential size of the entire dataset (Table 1). Additionally, Specimen 3 features rays formed by tracheid replacement with parenchyma cells, like those described in *Gmujij* (Pfeiler and Tomescu, 2021), alongside typical uniseriate rays (Table 1).

Specimen 6 is statistically similar to *Gmujij* in the variance and distribution of MI values (Fig. 10D) but is not closely comparable to the latter in the three types of regressions (Fig. 10A–C). *Gmujij* is also similar to Specimen 5 in the T*_n_* vs. T*_n_*_−1_ regression (Fig. 10A), but not in any of the other comparative metrics (Fig. 10B–D). The slight similarity of *Gmujij* to Specimens 5 and 6 is not unlike the similarity of *Gmujij* to *Franhueberia* (Fig. 8). Like its relationship to *Franhueberia*, *Gmujij* is different from both Specimen 5 and 6 in its mesarch actinostele that is significantly larger than the haplosteles of any of the new specimens or previously described taxa (Table 1); although the primary xylem of Specimens 5 and 6 are, at best, incompletely preserved (Figs 1A and 2D), the overall geometry of the specimens precludes the presence of an amount of primary xylem comparable to that of *Gmujij*. Additionally, *Gmujij* possesses tracheid-replacement rays, whereas Specimens 5 and 6 have typical uniseriate rays (Table 1). Thus, despite some aspects of similarity between Specimens 5 and 6 and *Gmujij*, consideration of the entire comparative dataset demonstrates that they are different, which is consistent with assignment of Specimens 5 and 6 to *Franhueberia*.

Specimens 1 and 2 are very similar in all the metrics, with the only difference emphasized in comparisons with *Armoricaphyton*. Specimen 1 is the only specimen showing variance and distribution of MI that are statistically similar to those of *Armoricaphyton* (Fig. 11D), which is different from all the other specimens in all our comparative metrics. Specimen 1 has, like *Armoricaphyton*, the largest tracheids in tangential direction of the entire dataset (Table 1). However, *Armoricaphyton* sits far from Specimen 1 in all three regressions (Fig. 11A–C), has tracheids that are double in maximum radial size compared to those of Specimen 1, and has a particular type of radial system that is unlike the typical uniseriate rays present in Specimen 1 (Table 1). As in the case of *Gmujij*, consideration of the entire comparative dataset rejects close similarity between Specimen 1 and *Armoricaphyton*.

## DISCUSSION

### Diversity of wood anatomy among the new fossil specimens and taxonomic implications

Our comparative metrics suggest that the six new specimens represent at least two and possibly three different woody taxa. Thus, Specimens 5 and 6 can be confidently assigned to *Franhueberia gerriennei* based on close quantitative similarity. Specimen 4 cannot be addressed conclusively in a taxonomic framework due to the lack of sufficient quantitative data. Specimens 1 and 2 stand out from Specimens 5 and 6, as well as from all previously described woody taxa, which suggests that they represent a new taxon, referred to here as Woody euphyllophyte sp. A. Specimen 3, showing limited similarity in comparative metrics to Specimens 1, 2 and 5 and to *Franhueberia*, but possesses *Gmujij*-type rays, unlike all of these (except for Specimen 5, which may also have *Gmujij*-type rays); consequently, it may represent a third taxonomically distinct entity among the six new specimens, referred to here as Woody euphyllophyte sp. B.

#### Franhueberia gerriennei

*Franhueberia gerriennei* is the first euphyllophyte with secondary growth identified in the Battery Point Formation (Hoffman and Tomescu, 2013). Until now, *Franhueberia* was only known from its type specimen. Thus, assignment of Specimens 5 and 6 to *Franhueberia gerriennei* contributes new data to characterize anatomical variation within this species. The type specimen of *F. gerriennei* lacks any extraxylary tissues. Therefore, the extraxylary tissues preserved in Specimen 5 contribute an additional layer of knowledge to this species and require emendation of the diagnosis. We also designate these two specimens as topotypes.

**Subdivision**: Euphyllophytina Kenrick and Crane, 1997
**Genus:** *Franhueberia* Hoffman and Tomescu, 2013
**Emended generic diagnosis** (emendations in bold type): Axes with centrarch protostele and secondary tissues produced by a vascular cambium. Metaxylem tracheids with circular to oval bordered pits; secondary xylem tracheids with multiaperturate scalariform bordered pits. Rays uniseriate. **Extraxylary tissues consisting of thick parenchymatous inner cortex and thin sclerified outer cortex**.
**Type species:** *Franhueberia gerriennei* Hoffman and Tomescu 2013
**Emended specific diagnosis** (emendations in bold type): Axis xylem **reaching >6 **mm in diameter. Protostele ca. 0.5 mm in diameter. Protoxylem tracheids 8 μm in diameter. Metaxylem tracheids 7–15 μm in diameter. Pitting round to oval, bordered, 4.4–6.6 μm in diameter. Secondary xylem tracheids rectangular in cross-section, 8.4–**37 **μm wide tangentially, 21.6–**42 **μm radially; scalariform bordered pits on tangential and radial tracheid walls, width same as that of tracheids, height 3.4–9.6 μm, separated by horizontal thickenings 1.4–3.4 μm across. Pit membranes with multiple apertures ca. 3 μm diameter, in one or two rows. Rays are more **or less** frequent, narrow (9–12 μm) and 90 μm to >150 μm tall. **Phloem unknown. Cortex thick, consisting of two sharply differentiated layers. Inner cortex parenchymatous, cells 15–90 μm. Outer cortex at least 95–130 μm thick, a mixture of thin- and thick-walled cells 12–46 μm. Outer cortex with undulating external outline due to shallow ridges and conical protrusions; protrusions 250–260 μm tall and 200–295 μm wide at base.**
**Topotypes**: One axis preserved in USNM 557840 Htop (b series slides) and one axis preserved in USNM 557840 Fbot c, Gtop b (G1top), Gbot (G1bot) and Htop e (c series slides in Fbot, b series slides in Gtop and G1top, and e series slides in Htop).

#### Woody euphyllophyte sp. A

This morphotype is represented by two specimens: one axis in USNM 557840 Cbot (a series slides) (Fig. 1B) and one axis USNM 557839-2b Atop (d series slides) (Fig. 1C). The primary xylem, preserved with distortions in only one of the specimens (Fig. 3A, B), forms a small centrarch haplostele with metaxylem tracheids up to 43 μm. The two specimens are characterized by significant thickness of wood with P-type tracheids (secondary xylem thickness 1.54 and 3.66 mm, respectively), numerous multiplicative divisions, and maximum tracheid sizes of 47 μm radially and 50 μm tangentially. Radial tracheid files flare centrifugally (MI) by ca. 2 μm per tracheid in tangential size (ranging roughly from 1.5 to 3.0 μm per tracheid; Supplementary Data Fig. S9D). Rays (Figs 3C, D and 5C–E) are uniseriate, probably parenchymatous. No extraxylary tissues are preserved.

#### Woody euphyllophyte sp. B

This morphotype consists of one specimen – an axis in USNM 557839-2b Bbot and Ctop (a series slides for both Bbot and Ctop) (Fig. 2A, B). The primary xylem of this specimen is a centrarch haplostele with metaxylem tracheids up to 28 μm in size tangentially and 59 μm in size radially that become elongated radially at the periphery of the primary xylem. Its wood is 0.5 mm in radius and consists of P-type tracheids with clear multiplicative divisions and maximum tracheid sizes of 40 μm radially and 45 μm tangentially. Radial tracheid files flare centrifugally (MI) by ca. 1.5 μm per tracheid in tangential size (deviations generally fall within 0.5 μm of this average; Supplementary Data Fig. S9D). Some of the rays appear similar to those seen in *Gmujij* (Fig. 4D, E), whereas others look more like typical uniseriate rays (Fig. 4F, G). Extraxylary tissues are incompletely preserved and highly distorted taphonomically (Fig. 2A, B). They suggest a parenchymatous layer of inner cortex containing around the stele scattered sclereids up to 32 μm in size with thick secondary walls, and a thin outer cortex consisting of large (up to 64 μm) sclerenchyma with thin secondary walls.

### Evo-devo implications of woody growth duration

The amounts of woody tissues present in the new specimens range between 0.47 mm of secondary xylem thickness in the smallest specimen and 3.66 mm in the largest. It is worth noting that the largest of the new specimens is surpassed in its amount of secondary xylem only by one other Early Devonian woody specimen, which was reported by Gensel (2018: plate III, figs 2 and 3). The second and third largest of the new specimens contain amounts of secondary xylem comparable to a few other large woody specimens reported by Gensel (2018). The comparatively large volumes of wood produced by all these specimens indicate that, by the end of the Emsian, secondary xylem had evolved in some euphyllophyte species to yield physiological or structural dividends that were worth the energy invested in building these tissues.

The thickness of the secondary xylem in the largest Early Devonian woody euphyllophytes also prompts speculation about their mode of secondary growth (*sensu*Tomescu and Groover, 2019), specifically, whether their cambia had unifacial or bifacial activity. Secondary phloem produced by a bifacial cambium has, among other roles, two specific functions. One is to substitute the primary phloem in its photosynthate conducting function. The other is to provide the living tissue from which the cork cambia that produce the canonical periderms (*sensu*Lalica and Tomescu, 2024) of the bark can be specified, once enough secondary growth has happened. Both of these roles are underpinned by mechanical effects of the increase in secondary xylem volume as a result of continued growth from the cambium. The addition of girth beneath the cambium, in the form of secondary xylem, (1) induces tensional stress in all the tissues external to the cambium, while also (2) compressing those tissues between the ever-expanding cambium and the outermost layers (epidermis, outermost cortex layers), which do not compensate for the internal increase in girth by corresponding growth responses. Both of these effects would eventually obliterate the tissues outside the cambium, unless they are compensated for by production of secondary phloem, which (1) replaces the primary phloem that is compressed beyond functionality and (2) provides a stock tissue for production of periderms that replace the outermost tissues torn by tensional stresses.

The corollary of these functions of the secondary phloem is that absence of this tissue, in the case of plants with a unifacial vascular cambium, may place limitations on the amount of secondary xylem a plant can produce from that cambium. From the perspective of periderm production, observations on living plants suggest that this structural layer, whose formation does not involve the secondary phloem initially, typically starts after considerably more secondary xylem has been produced than the volumes observed in even the largest Early Devonian woody euphyllophytes. Thus, the ‘periderm constraint’ on presence of a bifacial cambium probably requires larger volumes of wood than those documented in Early Devonian plants. In other words, given the still small maximum thicknesses of secondary xylem documented in Early Devonian euphyllophytes, presence or absence of canonical periderm in these plants has no bearing on the mode of cambial growth. As an aside on periderm, it is worth noting that the only evidence of periderm production in Early Devonian euphyllophytes consists of patches of wound periderm produced in axes that had not undergone any vascular secondary growth (Banks, 1981; Banks and Colthart, 1993; Lalica and Tomescu, 2023).

The ‘extraxylary tissue compression constraint’, on the other hand, allows for speculation that the largest Early Devonian woody plants documented to date may have had bifacial vascular cambia. While unifacial cambia are unknown among extant plants, they have been demonstrated in the extinct zygopterid ferns, where they produced secondary xylem but no secondary phloem (Dennis, 1974). If extraxylary tissue compression placed a constraint on the amount of secondary xylem production, then secondary growth would have been determinate in the zygopterids and ceased upon reaching an upper size limit (as suggested by Cichan and Taylor, 1990), in the same way it does in the sphenophyllalean sphenopsids (although for a different reason – absence of multiplicative divisions; Cichan and Taylor, 1982; Cichan, 1985*b*). Under this hypothesis, the largest woody stems documented in zygopterids would provide a maximum secondary xylem thickness beyond which secondary growth could have been sustained only if arising from a bifacial vascular cambium. The thickness of the secondary xylem in the largest zygopterid stems with secondary growth (Dennis, 1974; Phillips and Galtier, 2005) does not exceed 1.7 mm. This thickness is significantly smaller than that of the secondary xylem of *Franhueberia* and Woody euphyllophyte A, as well as those of several woody specimens documented by Gensel (2018), which implies that all those plants may have had bifacial cambia. On the other hand, it is also possible that lepidodendralean lycopsids and calamitalean sphenopsids possessed unifacial vascular cambia with determinate growth (discussed in Cichan and Taylor, 1990; see also D’Antonio and Boyce, 2021; D’Antonio, 2023). If so, the much larger amounts of secondary xylem – compared to those of zygopterids or the Early Devonian woody euphyllophytes – documented in some of their representatives (e.g. Frankenberg and Eggert, 1969; Rößler *et al.,* 2012) do not support the speculations based on zygopterids. Irrespective of these discussions, while secondary phloem has not been demonstrated up until now in any Early Devonian plant, it is possible that some Early Devonian euphyllophytes produced secondary phloem. Because bifacial cambia are known only in the lignophyte clade (Crane, 1985; Doyle and Donoghue, 1986), for which they are a synapomorphy, presence of bifacial cambia in Early Devonian euphyllophytes would either imply independent evolution of bifacial cambia in different euphyllophytes or would push the age of lignophytes down into the Early Devonian. Thus, the search for plants preserving secondary phloem in the Early Devonian continues.

### A new method of quantifying and comparing wood anatomy

A number of anatomical and developmental variables and constraints – discussed in every plant anatomy textbook (e.g. Esau, 1965; Fahn, 1990) – interact to determine the size of tracheids in the secondary xylem. One of these is the tangential size of cambial initials, which dictates the tangential size of the tracheids they produce by periclinal (additive) divisions. The tangential size of cambial initials increases with successive periclinal divisions that lead to expansion of the cambium, until they reach a maximum size that triggers – possibly in response to tensional tangential stress – multiplicative (anticlinal) division, which resets the size of cambial initials. Another variable is the duration of growth in radial dimension of maturing tracheids prior to the onset of differentiation processes that stop cell growth – the shorter their growth interval, the smaller the radial size of mature tracheids. Additionally, the nature of plant multicellularity, which involves cells firmly and permanently attached on all sides to other cells, imposes mechanical constraints on the variables mentioned earlier, as do the presence and amount of coordination of the cell growth and differentiation processes between the cells that are physically attached to each other.

The fact that the wood of a species has consistent anatomy among individuals of that species is a good indication that the mechanisms that control all the variables listed above must be consistent and tightly canalized within each species. Thus, wood anatomy can provide qualitative criteria for comparing and identifying species (e.g. Phillips, 1979; Wheeler *et al.*, 1989; Richter *et al.*, 2004). Moving beyond qualitative comparisons to develop quantitative criteria for comparing the secondary xylem of different taxa is delicate and has been generally avoided, due to the many external and internal factors (some of them listed above) that determine various aspects of wood anatomy and result in variations between individuals of a species and even within individual plants. Despite these problems, it may be possible to quantify some aspects of wood anatomy in ways that capture and emphasize those features that vary within narrow ranges in a species and differentiate between species. An anatomical parameter that results from the intersection of the variables and constraints discussed above is tracheid size as a function of its position along the radial axis of secondary xylem. This parameter can be quantified by different metrics, some of which were developed by Cichan (1985*a*) and D'Antonio (2023). We tested a number of metrics that relate tracheid size and position in the developmental sequence of secondary xylem, for their breadth of variation within taxa and their effectiveness in separating taxa, at the genus level. Our comparisons that involved extant gymnosperm genera and previously described extinct species representing different genera identified four metrics that provide the most conclusive comparisons: (1) the tangential size of each secondary xylem tracheid (measured on its outer periclinal wall) as compared to that of the tracheid immediately preceding it developmentally; (2) the tangential size of each secondary xylem tracheid as a function of the absolute distance of its outer periclinal wall from the primary xylem; (3) the increase in tangential size of each tracheid (between its outer and inner periclinal walls) per unit of radial size of that tracheid; and (4) the mean increase in tangential size per tracheid along a radial file of tracheids.

This comparative approach using the four metrics is not without caveats. One is that none of the four metrics, by itself, separates reliably all the taxa. Instead, each of the four metrics separates more clearly some of the taxa from the others. Because the more obviously separated taxa are different for each metric, the four metrics need to be used in concert to reach the most conclusive comparative results. A second caveat, which may be in part underpinning the previous one, is that at least those metrics that involve radial tracheid sizes are influenced (albeit to unknown or unmeasurable extent) by environmental variables. To minimize this, one has to ideally avoid measuring tracheid series that represent long developmental intervals, which are more likely to be significantly affected by seasonality (e.g. early wood – late wood differences in radial tracheid size). This means that the method operates most reliably with short tracheid series of 10–12 tracheids and probably fewer than 15 tracheids, that are not affected by multiplicative (anticlinal) divisions. A third caveat is that only one of the four metrics (the mean increase in tangential size per tracheid along a radial file of tracheids) allows for statistical tests of similarity between taxa. The other three are regressions for which methods of pairwise statistical comparison do not exist, so they can only be compared visually. In this respect, the method we present here is only opening a path that could and should be extended and broadened by future searches for additional metrics and methods to statistically compare secondary xylem anatomy. A fourth caveat is associated with the topic of additional methods of comparison. Namely, we do not know whether and how well the metrics that we identified as working well together to separate taxa at the genus level also work in separating different species within a genus or, more generally, taxa at different phylogenetic depths. Certainly, more exploratory data collecting and analysis to address these questions is warranted and could make the subject of future studies.

Due to the above caveats, the method presented here may not provide unequivocal answers in all instances. However, it provides the first tool developed to date for comparing quantitatively plant fossils that are incompletely preserved, even to the point of consisting exclusively of wedges of secondary xylem. The above caveats, as well as the examples presented here, also indicate that the conclusiveness of this method is ideally complemented towards complete elimination of equivocation by the integration of metrics with data on even just a few additional anatomical features such as the stele type or the type of radial tissue system present in the secondary xylem.

## CONCLUSIONS

Six new specimens of Early Devonian woody plants inspired a search for metrics to quantify their wood anatomy in ways that are relevant to taxonomic comparisons. Investigation of extant gymnosperms and of previously recognized Early Devonian woody taxa provided quantitative datasets that led to the selection of four wood anatomy metrics that allow for distinguishing between taxa at the genus level based on the variation of tracheid size as a function of position in the secondary xylem. The four metrics developed for the new method presented here are most effective when used in conjunction with each other and in combination with other features of xylem anatomy.

Applied to the new woody fossils investigated here, our new method of comparison based on secondary xylem metrics led to identification of two new specimens of the previously described euphyllophyte *Franhueberia gerriennei* and to recognition of two putative new taxa of woody euphyllophytes. The new *Franhueberia* specimens contribute information of extraxylary tissues and expand the size range of the axes. The two putative new taxa require further investigation and additional specimens for formal taxonomic treatment; one of them exhibits relatively extensive woody growth, whereas the other has some extraxylary tissues preserved. These new specimens, along with previously reported woody plants, paint a picture of Early Devonian secondary growth that includes broader taxonomic diversity than previously recognized. This diversity – currently including five formally described genera and five other taxonomically distinct but not formally described types – reflects a significant interval of evolution of secondary growth and diversification of woody plants prior to the Emsian. These plants encompass anatomical diversity in terms of the radial tissue systems included in their secondary xylem [Pfeiler and Tomescu (2023) and this study] and include specimens with extensive development of wood [Gensel (2018) and this study]. The latter specimens suggest that some Early Devonian woody plants could have possessed bifacial vascular cambia that also produced secondary xylem.

As the scope of this study was largely concerned with the taxonomic placement of new fossil specimens, the development of the method we presented here opens up interesting opportunities and avenues for future work. Aside from formal treatments of the new woody types reported here, future work is needed to (1) identify additional metrics of secondary xylem anatomy that can effectively distinguish taxonomic signals; and (2) test additional ways of using in statistical comparisons the types of measurements we used (e.g. employing multivariate approaches). These should be done in concert with sampling of additional extant taxa (within and between species, genera, etc.) and increase of sample sizes within each taxon to test the accuracy and robustness of these comparative metrics at different taxonomic levels and phylogenetic depths. In terms of the metrics we used here, it will be helpful to develop methods for quantitative (rather than visual) comparisons of regression equations, possibly by using machine learning. Certainly, the method we developed here should be applied to other Early Devonian woody plants, such as those described by Gensel (2018) and other woody specimens identified in the Battery Point Formation.

## Supplementary Material

mcaf122_Supplementary_Data

## SUPPLEMENTARY DATA

Supplementary data are available at *Annals of Botany* online and consist of the following. Figure S1. Notations for formula used to calculate corrected measurements for disrupted tracheids. Figure S2. Ratio of tangential tracheid size (T*_n_*) relative to that of the first tracheid in the file (T_1_) for extant genera. Figure S3. Ratio of tangential size between each tracheid (T*_n_*) and the one preceding it in the file (T*_n_*_−1_) for extant genera. Figure S4. Ratio of tangential tracheid size (T*_n_*) relative to that of its radial size (R*_n_*) for extant genera. Figure S5. Ratio of relative increase in tangential tracheid size (RIT*_n_*) relative to the radial size of the tracheid (R*_n_*) for extant genera. Figure S6. Ratio of the relative increase in tangential tracheid size RIT*_n_* compared to that of the previous tracheid in the file (RIT*_n_*_−1_) for extant genera. Figure S7. Effect of the sampling of different tracheid sequences within a file in extant taxa, for the ratio of tangential size between each tracheid (T*_n_*) and the one preceding it in the file (T*_n_*_−1_) and for tangential tracheid size (T*_n_*) against cumulative R. Figure S8. Effect of the sampling of additive sets of measurements along tracheid files in extant taxa, for the ratio of tangential size between each tracheid (T*_n_*) and the one preceding it in the file (T*_n_*_−1_) and for tangential tracheid size (T*_n_*) against cumulative R. Figure S9. Comparisons of new woody specimens from the Battery Point Formation based on multiple metrics. Supplementary Data Sheet S1. Tracheid measurements in extant taxa, fossil taxa and new specimens. Supplementary Data Sheet S2. Tracheid files measured in each specimen. Supplementary Note. Regression equations for all taxa and metrics.
