# Supplementary material for: Characterizing and distinguishing the earliest woody euphyllophytes based on secondary xylem anatomy: method development and application
Source: Ann Bot. 2025 Jun 13;137(6):1602–23. doi: 10.1093/aob/mcaf122 (PMC13274980; doi:10.1093/aob/mcaf122)
Supplement: mcaf122_Supplementary_Data [file mcaf122_supplementary_data.zip › CasselmanTomescu2024_SupplementaryDataSheet2.pdf]

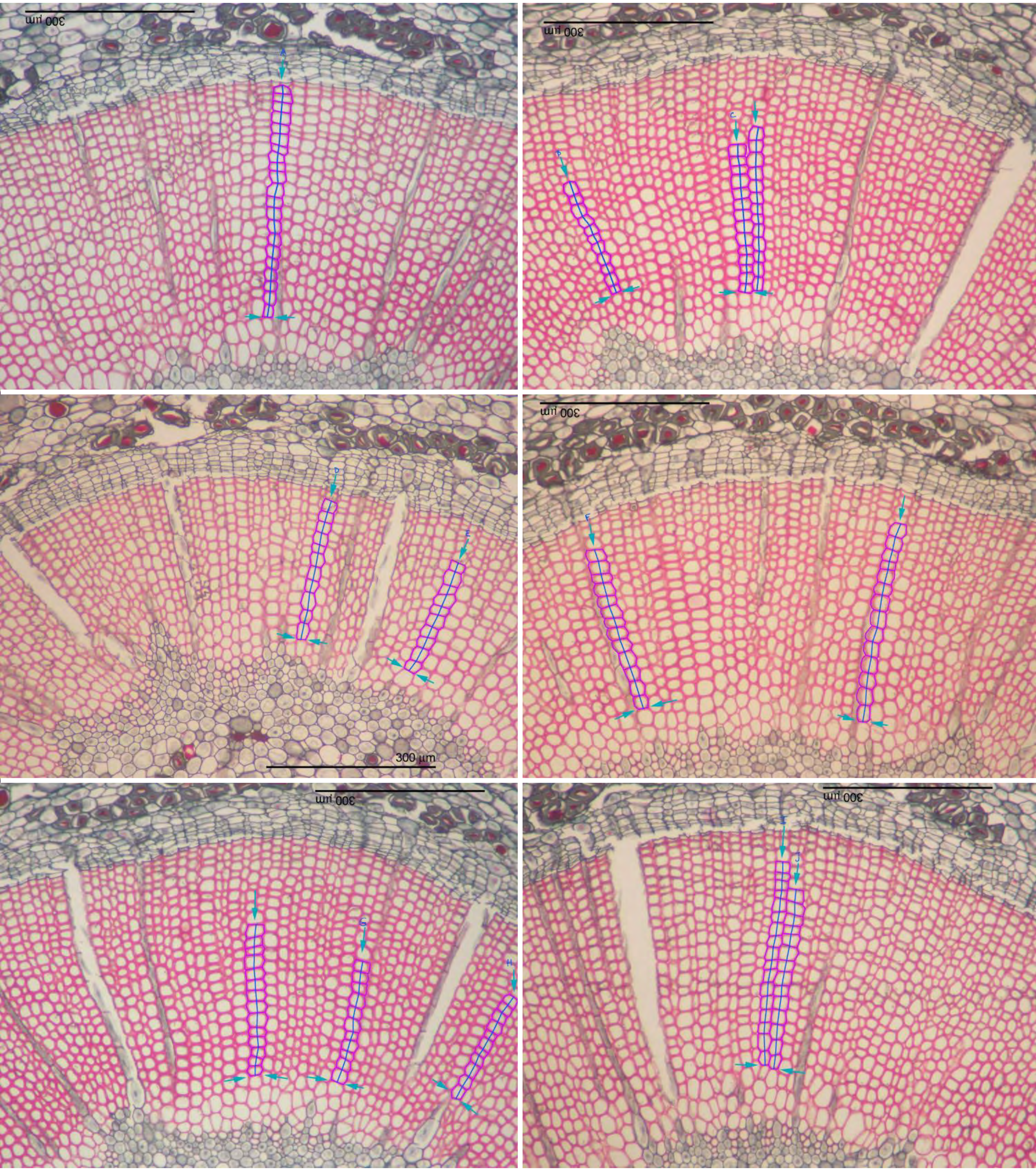

Ginkgo stem

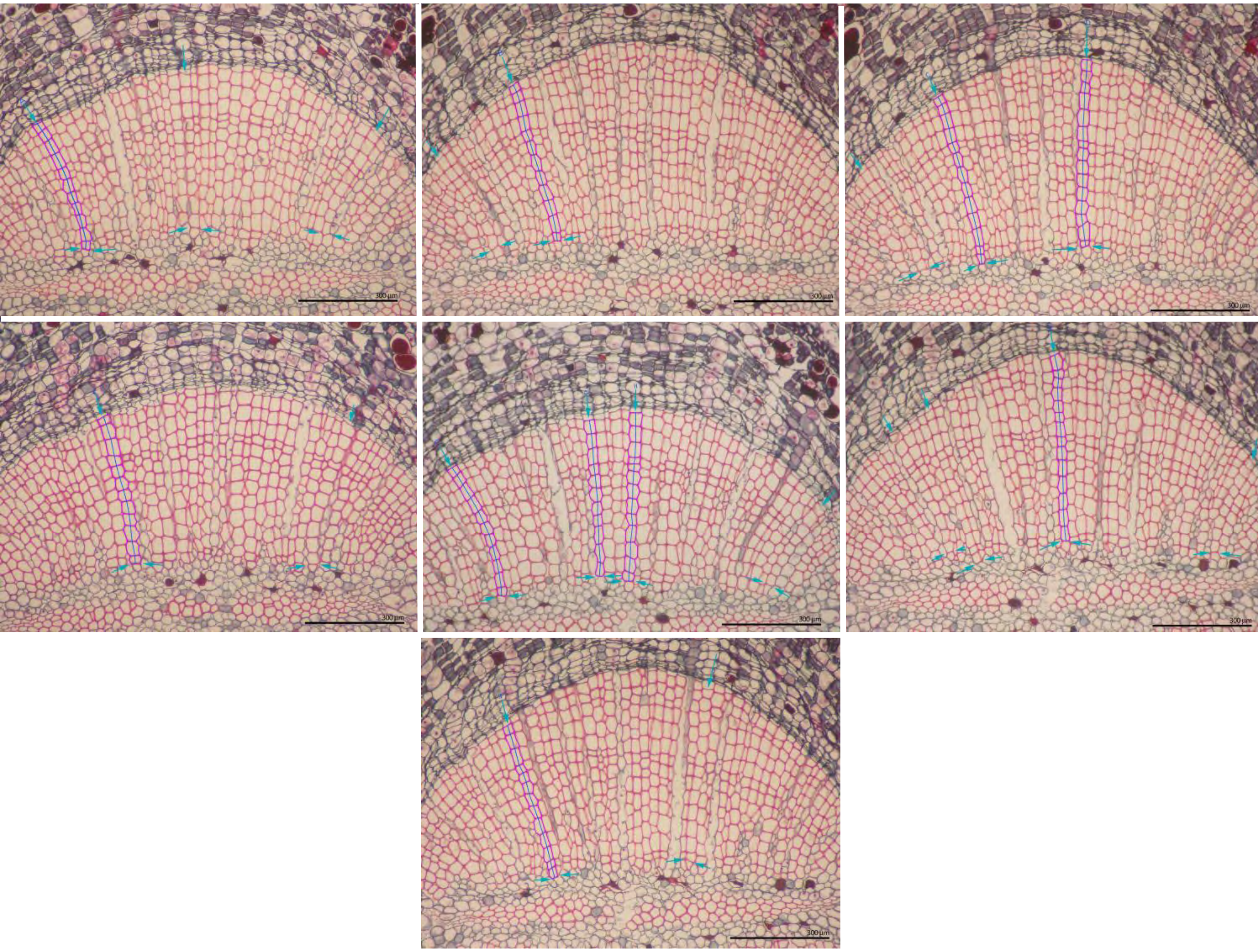

Ginkgo root

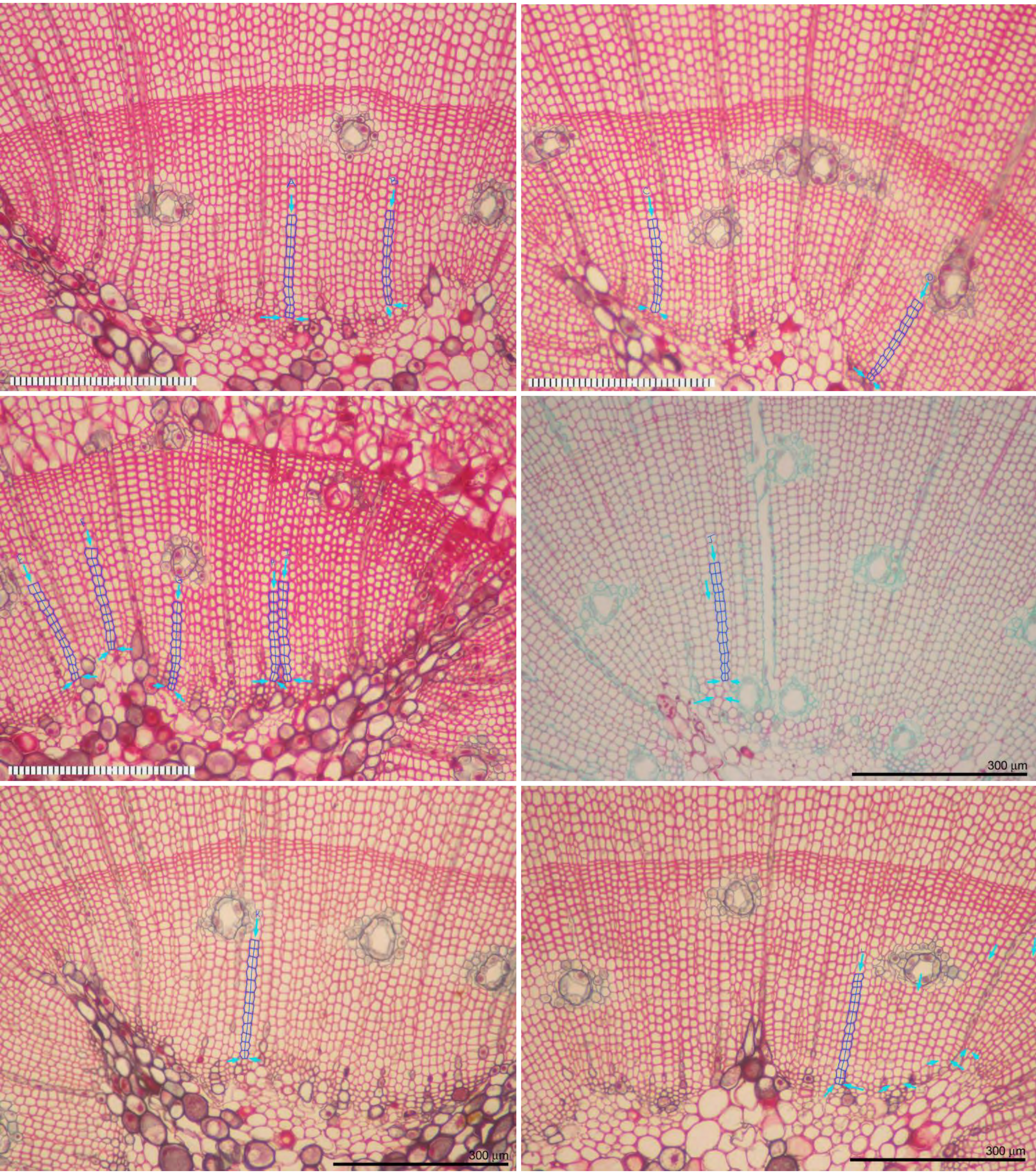

Pinus stem

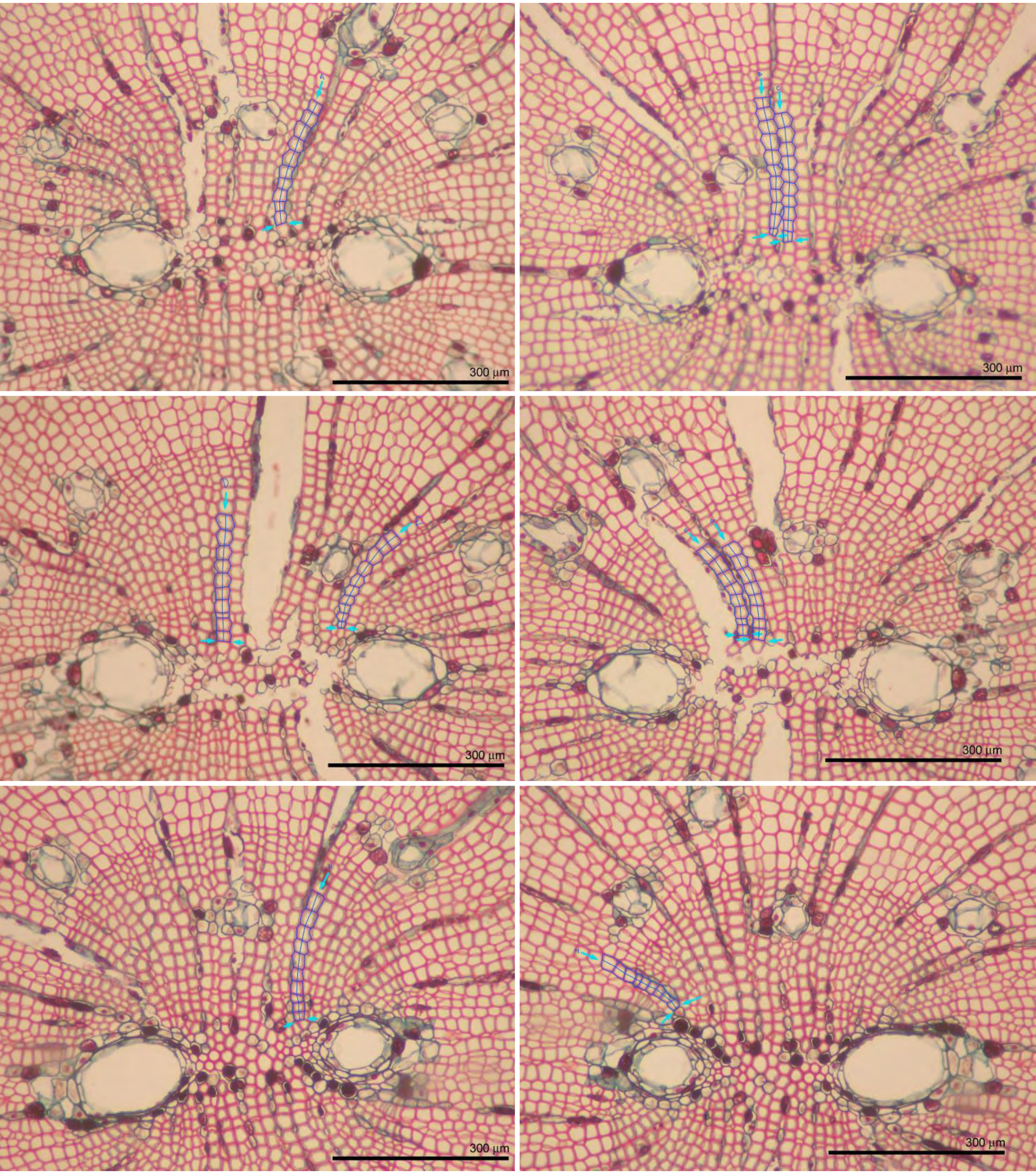

Pinus root

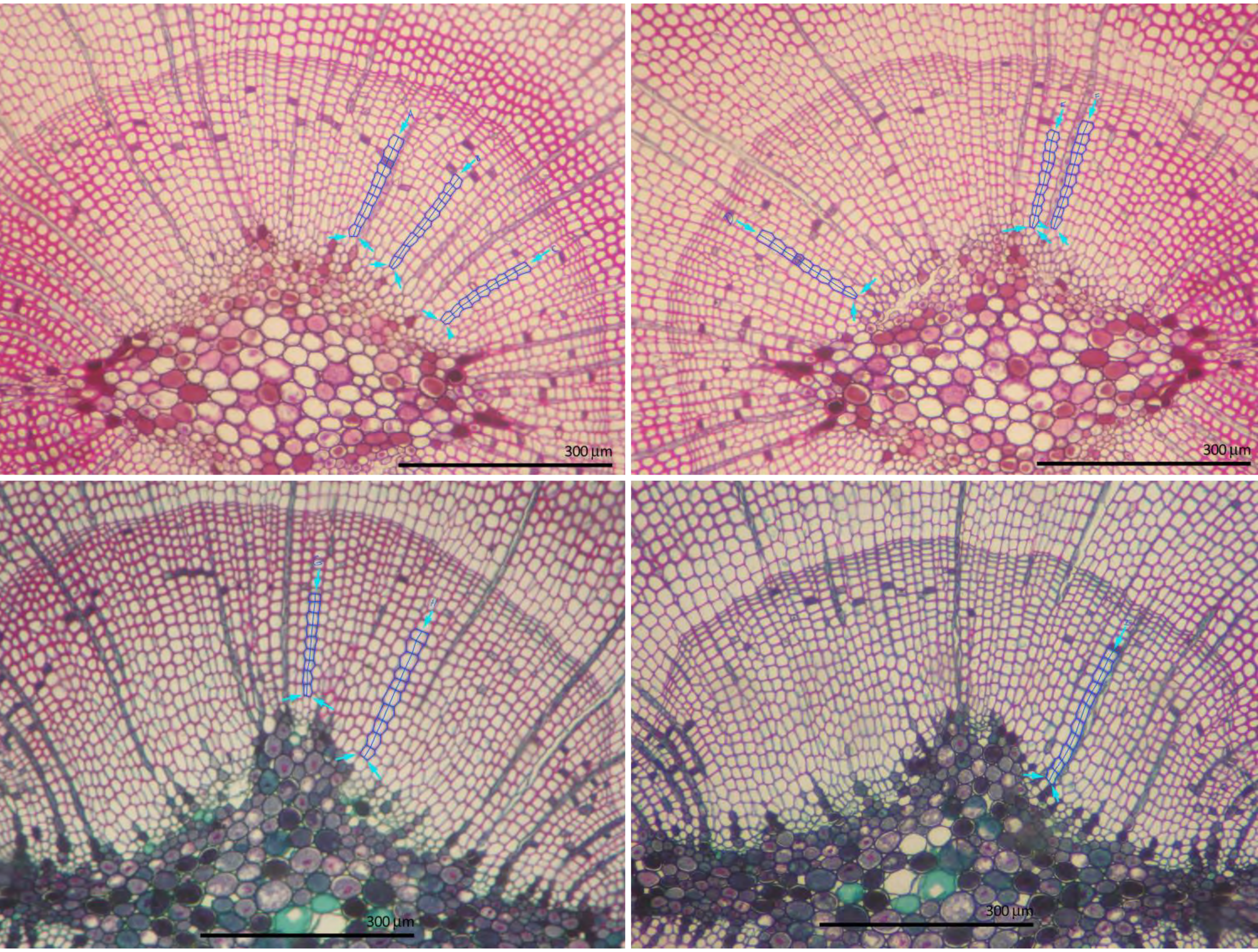

Sequoia stem

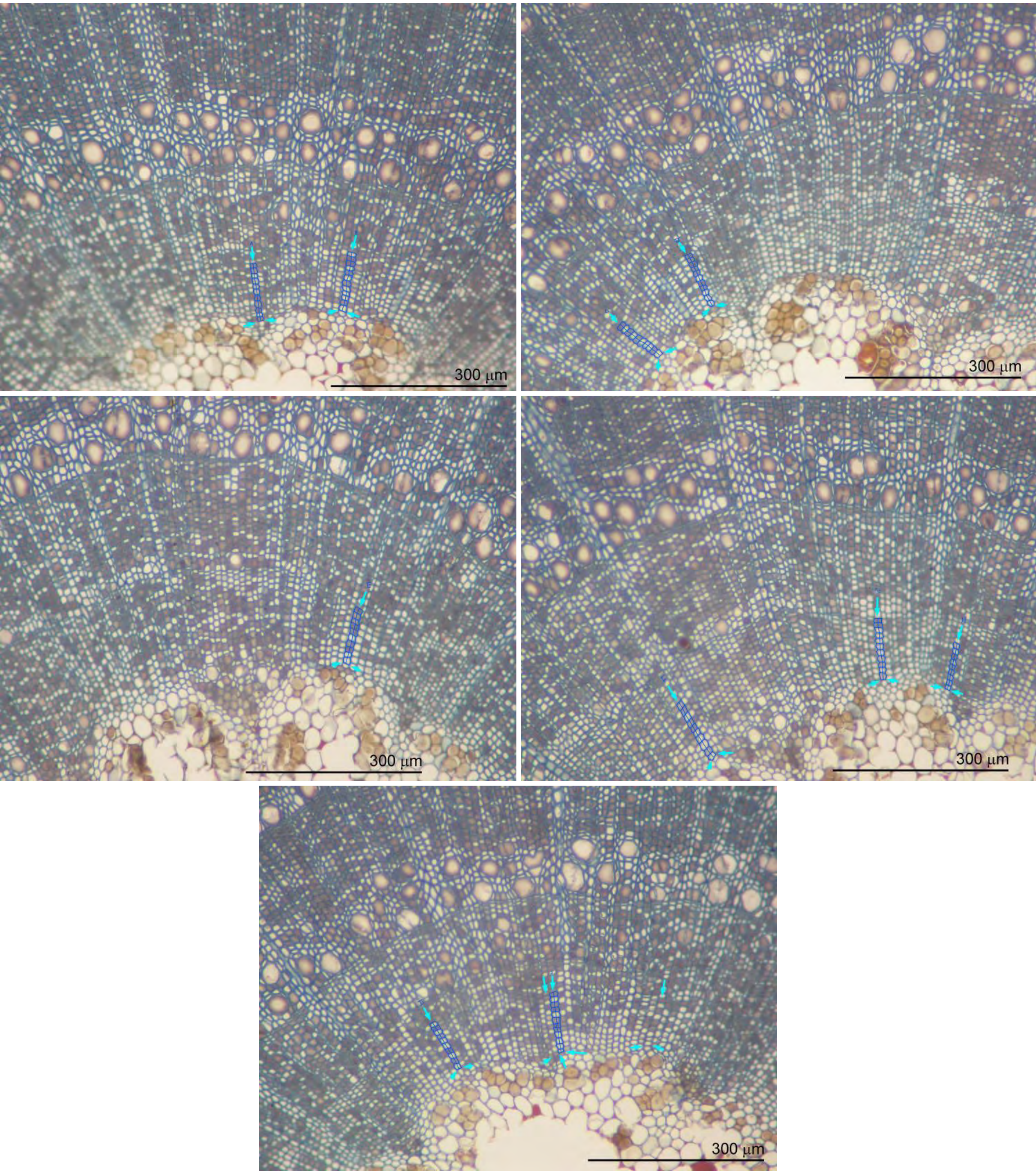

Ephedra stem

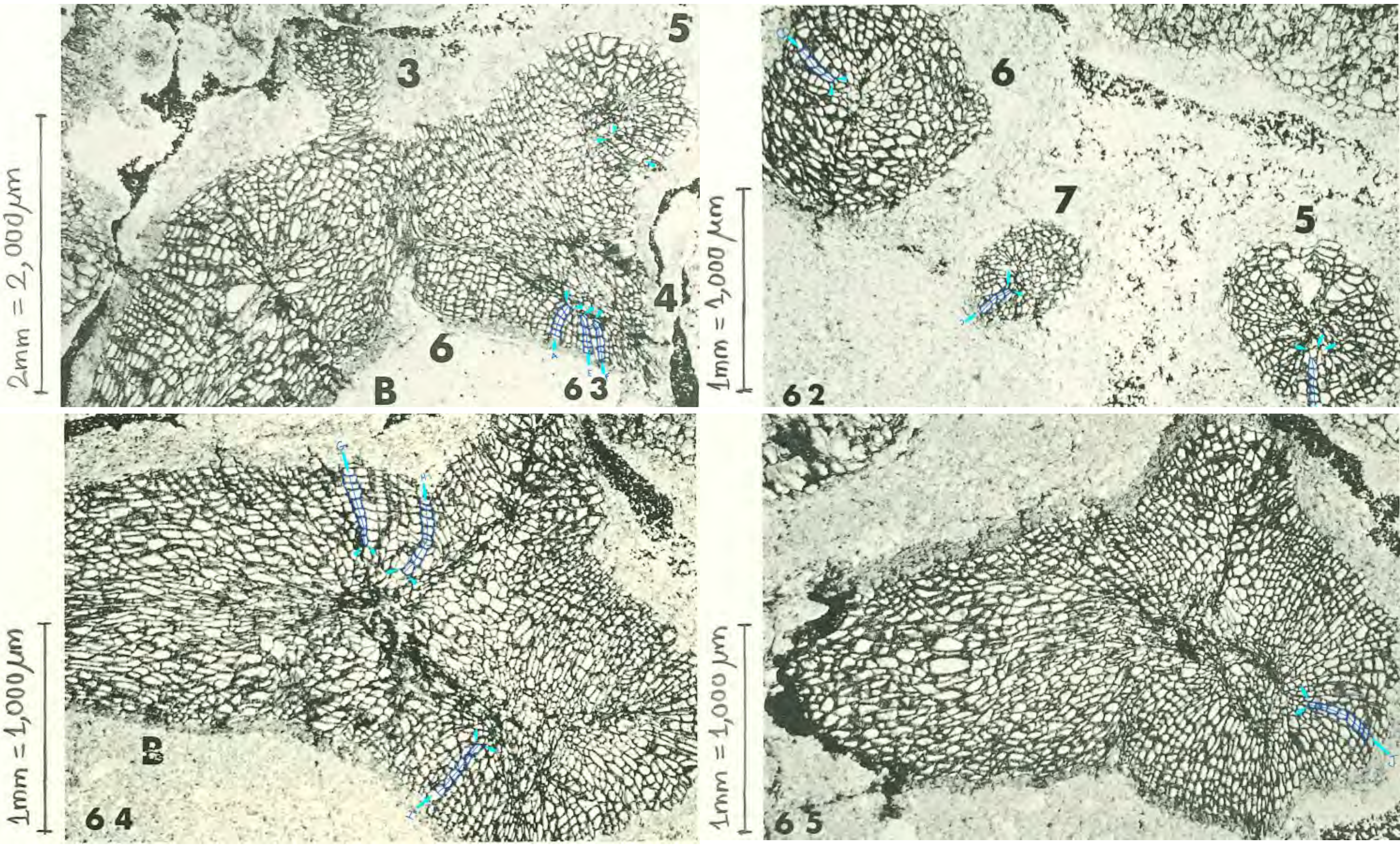

Psilophyton dawsonii (from Banks *et al.*, 1975)

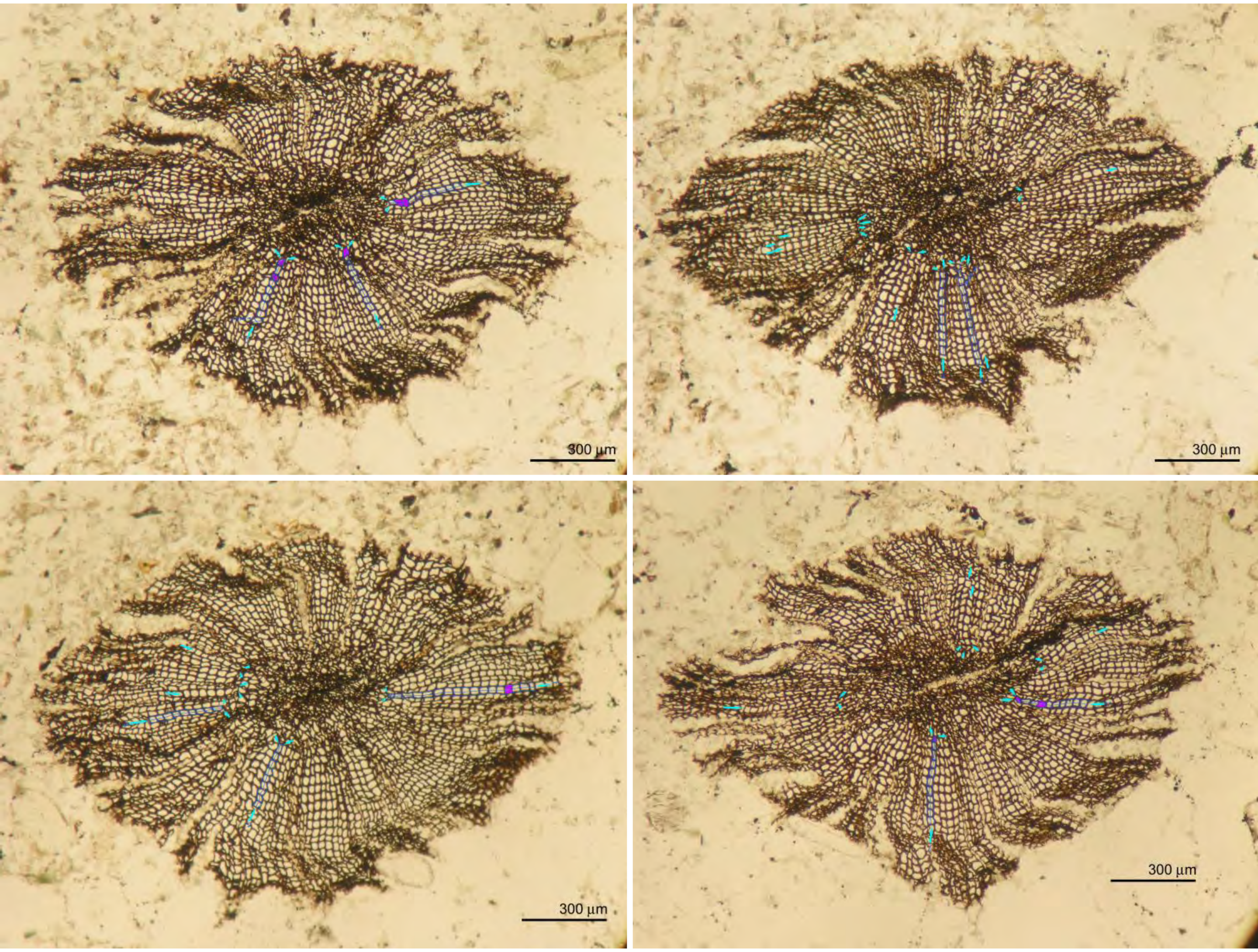

Franhueberia gerriennei

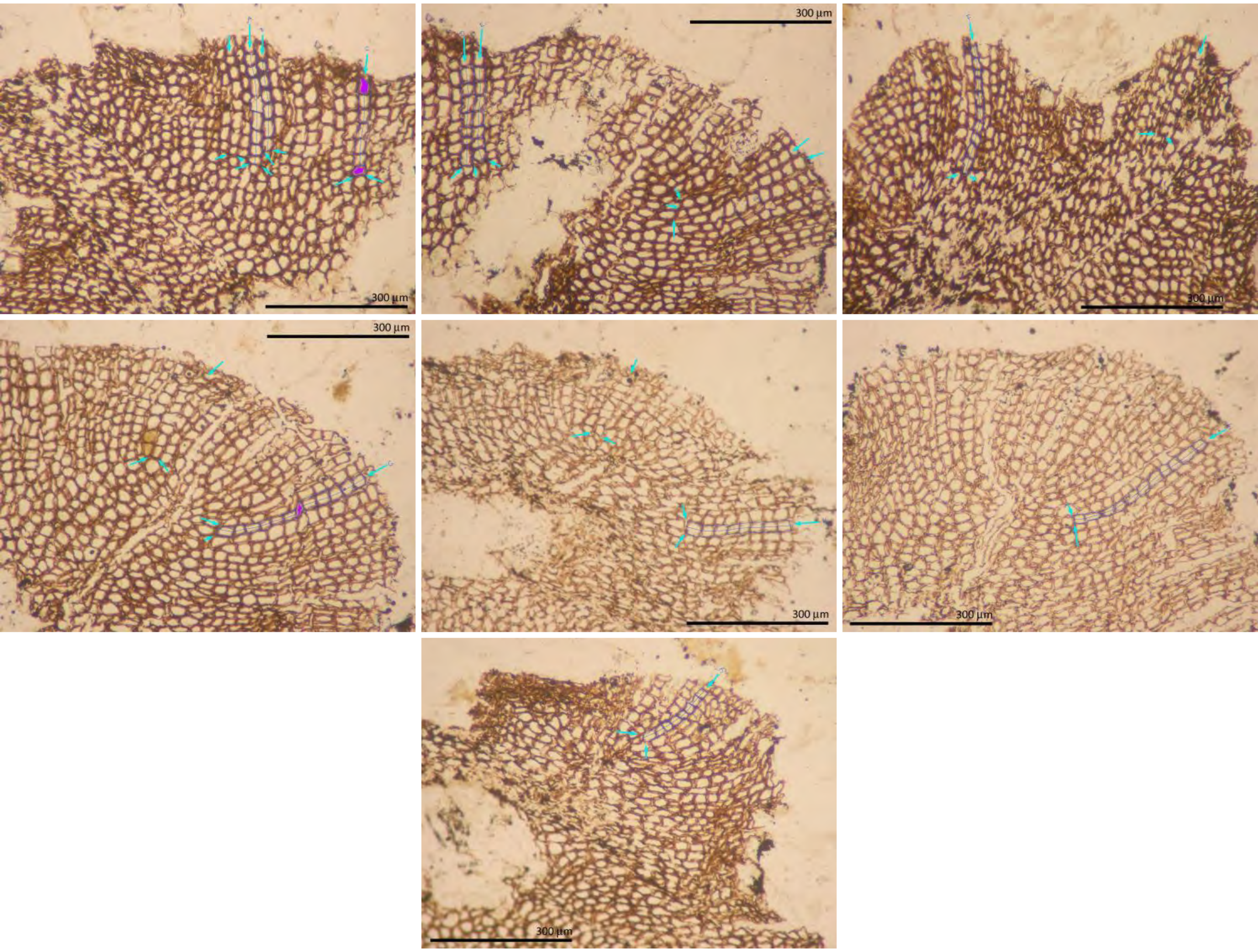

Gmujij tetraxylopteroides

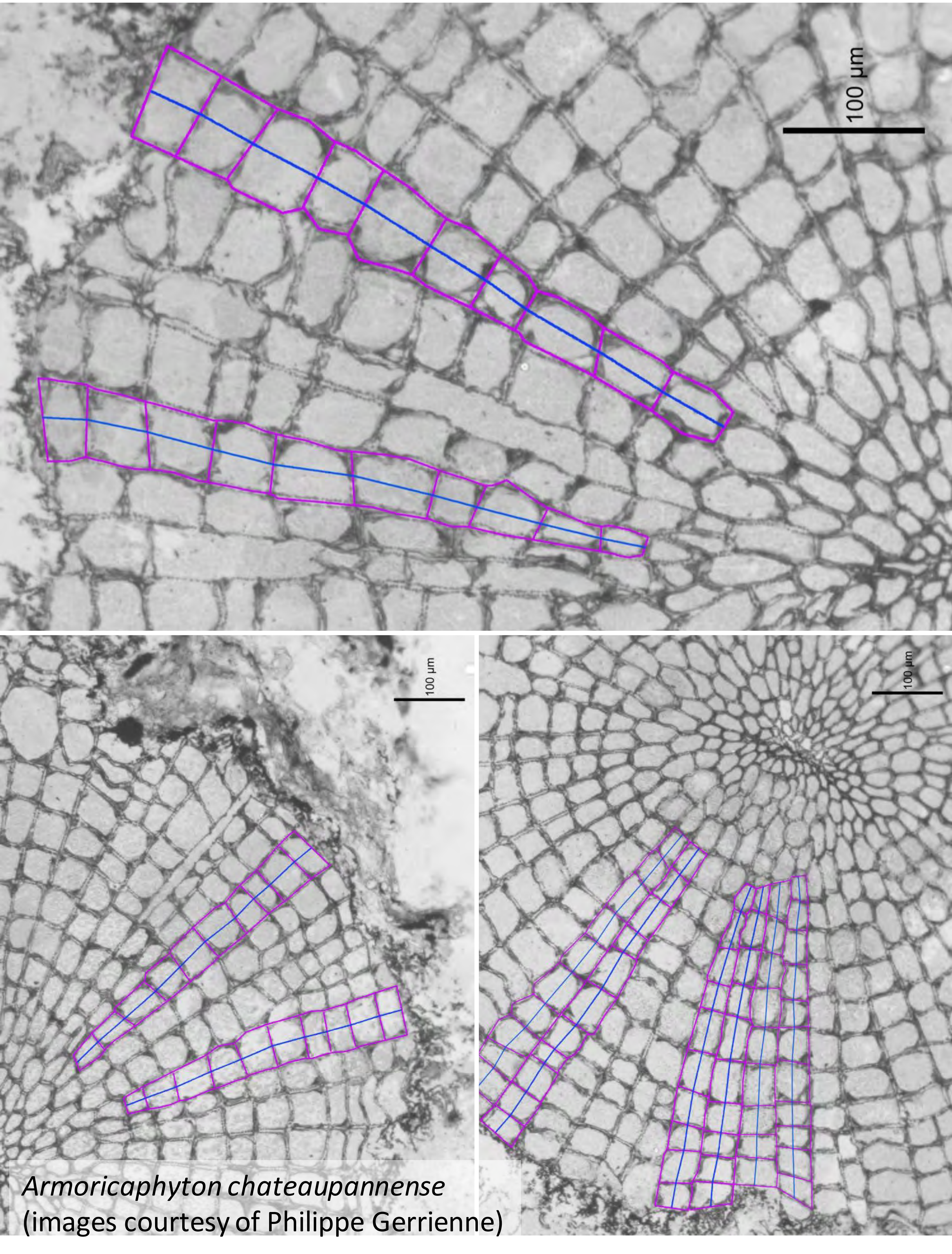

Armoricaphyton chateaupannense
(images courtesy of Philippe Gerrienne)

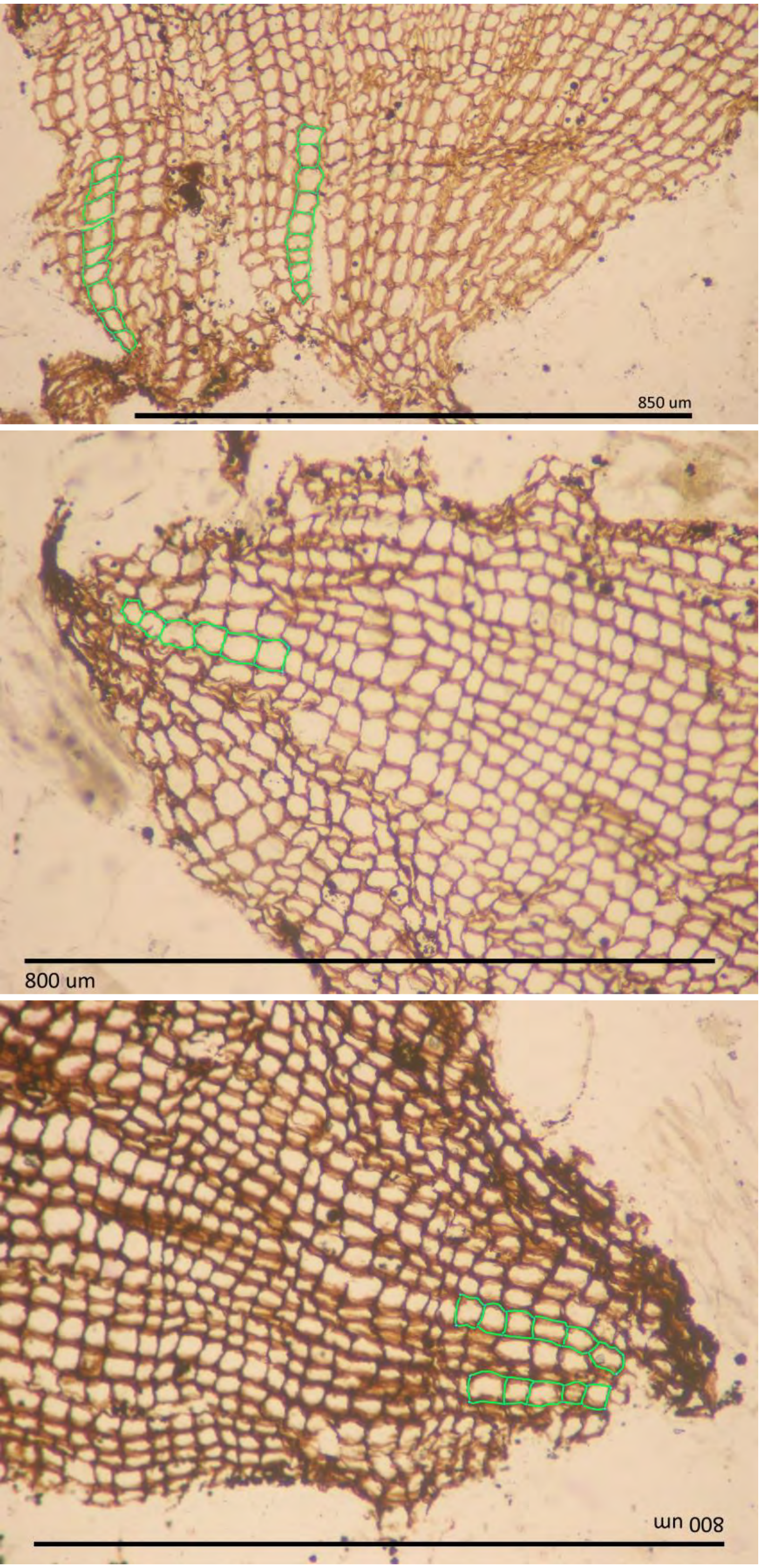

Specimen 1
557840 Cbot a

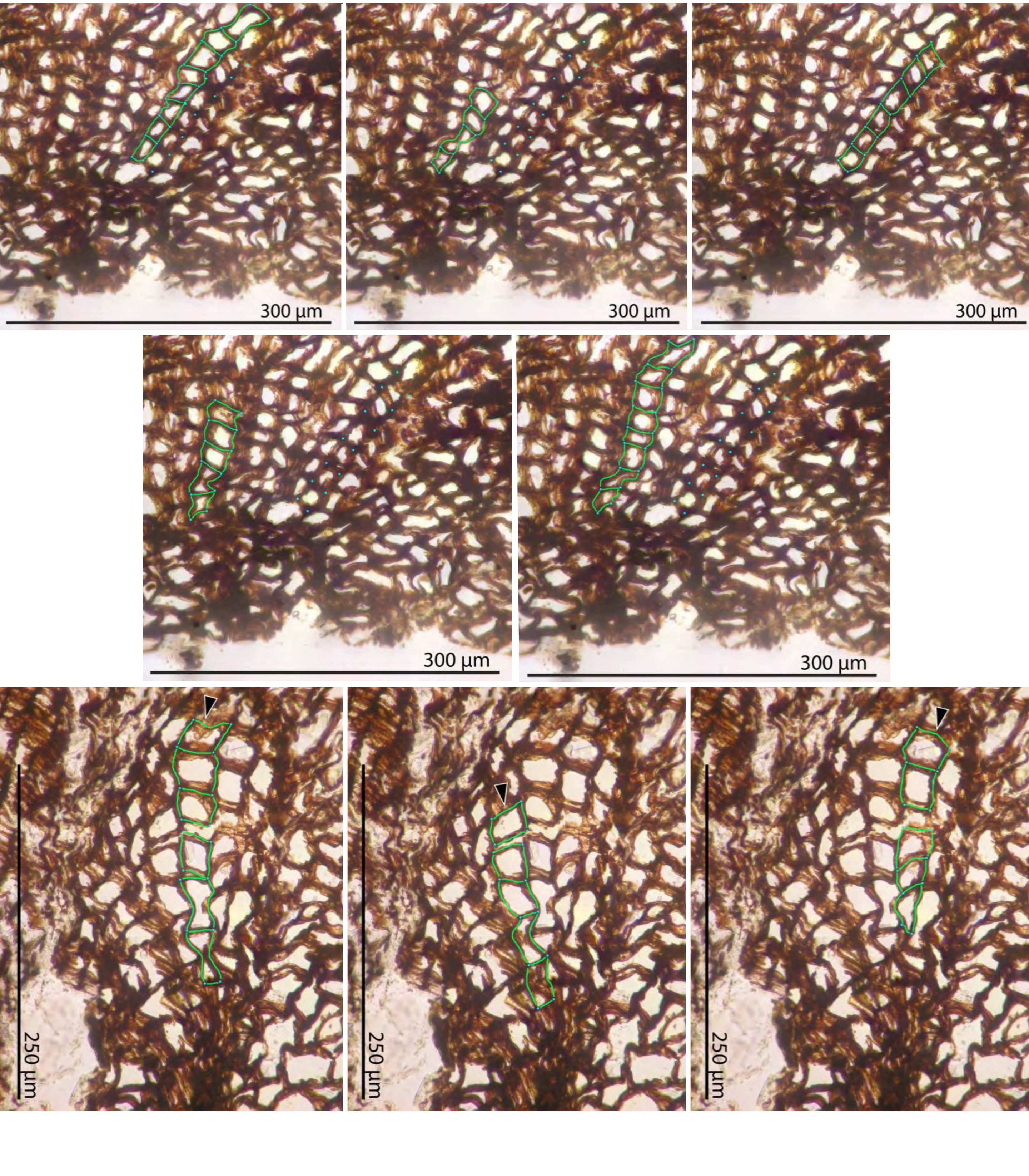

Specimen 2
557839-2b Atop d

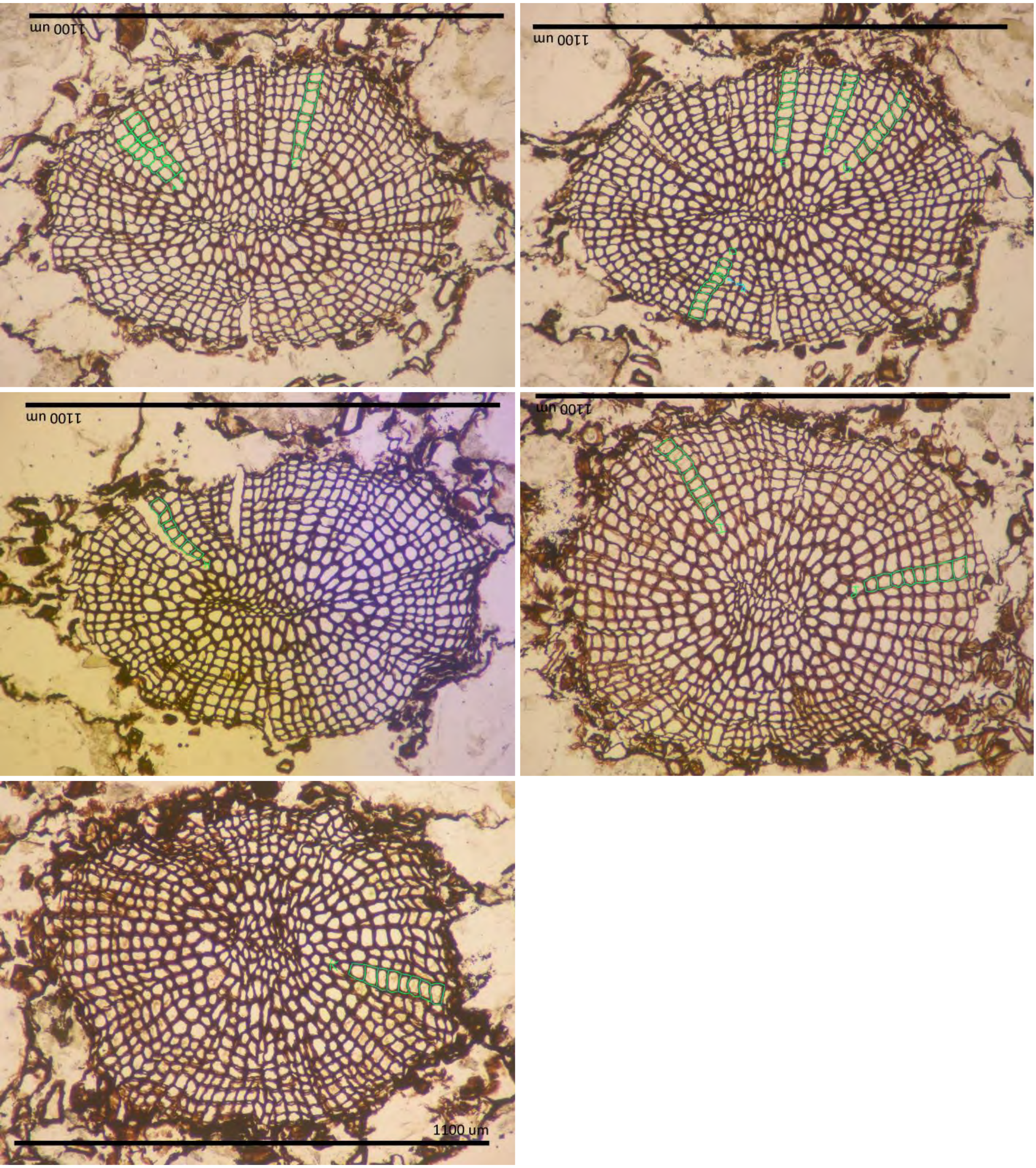

Specimen 3
557839-2b Bbot a

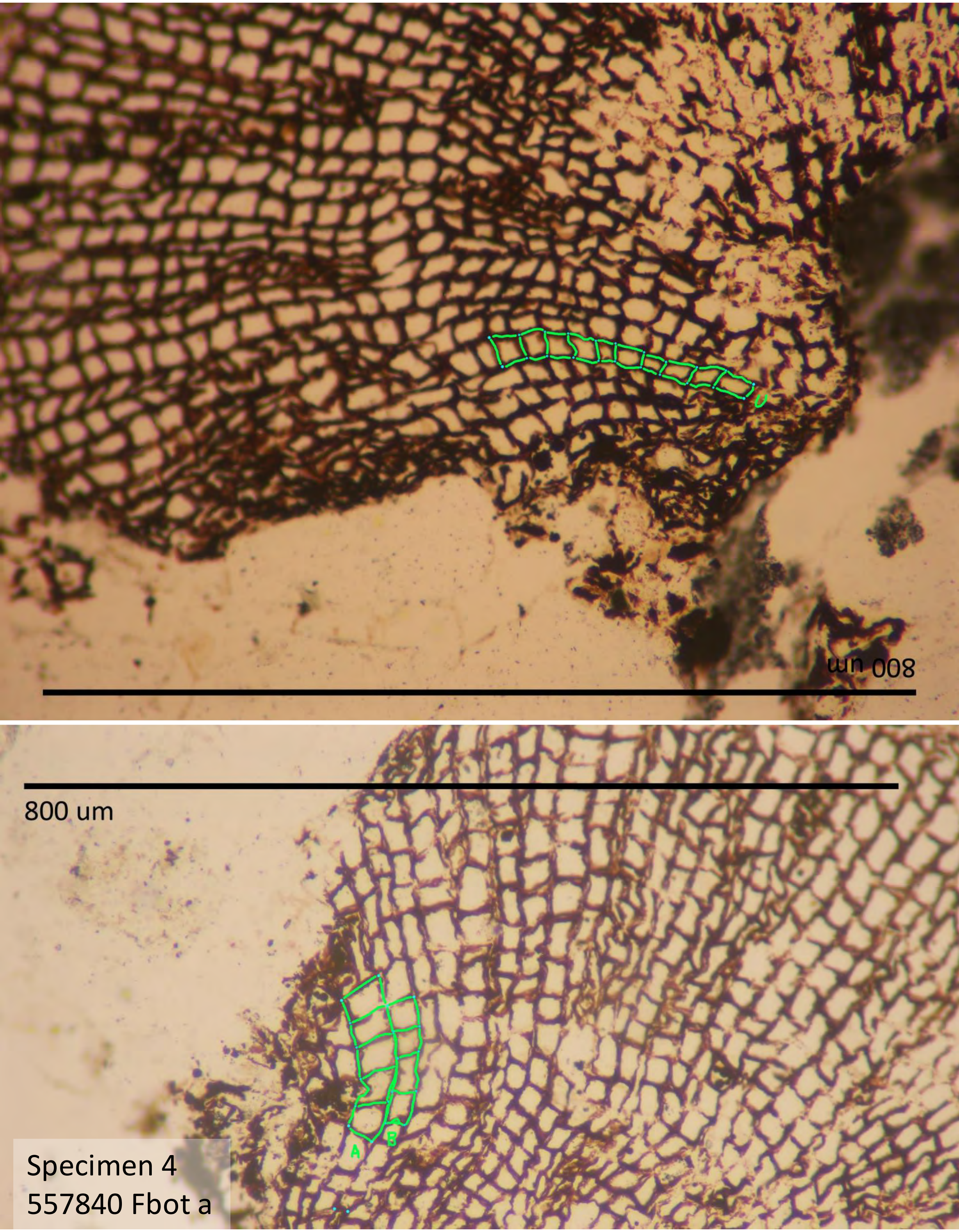
800 um
800 um
A
B
Specimen 4
557840 Fbot a

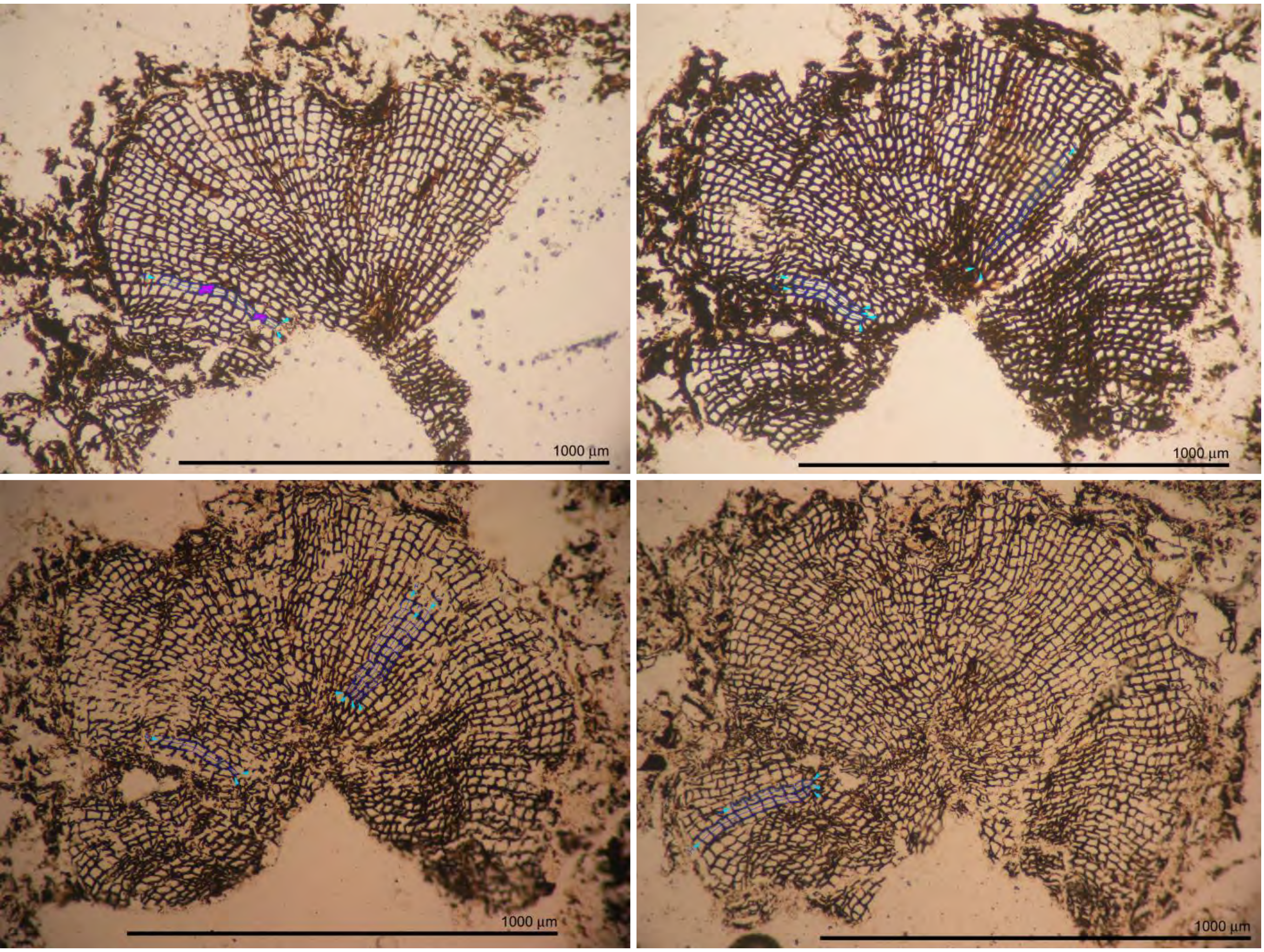

Specimen 5
557840 Htop b

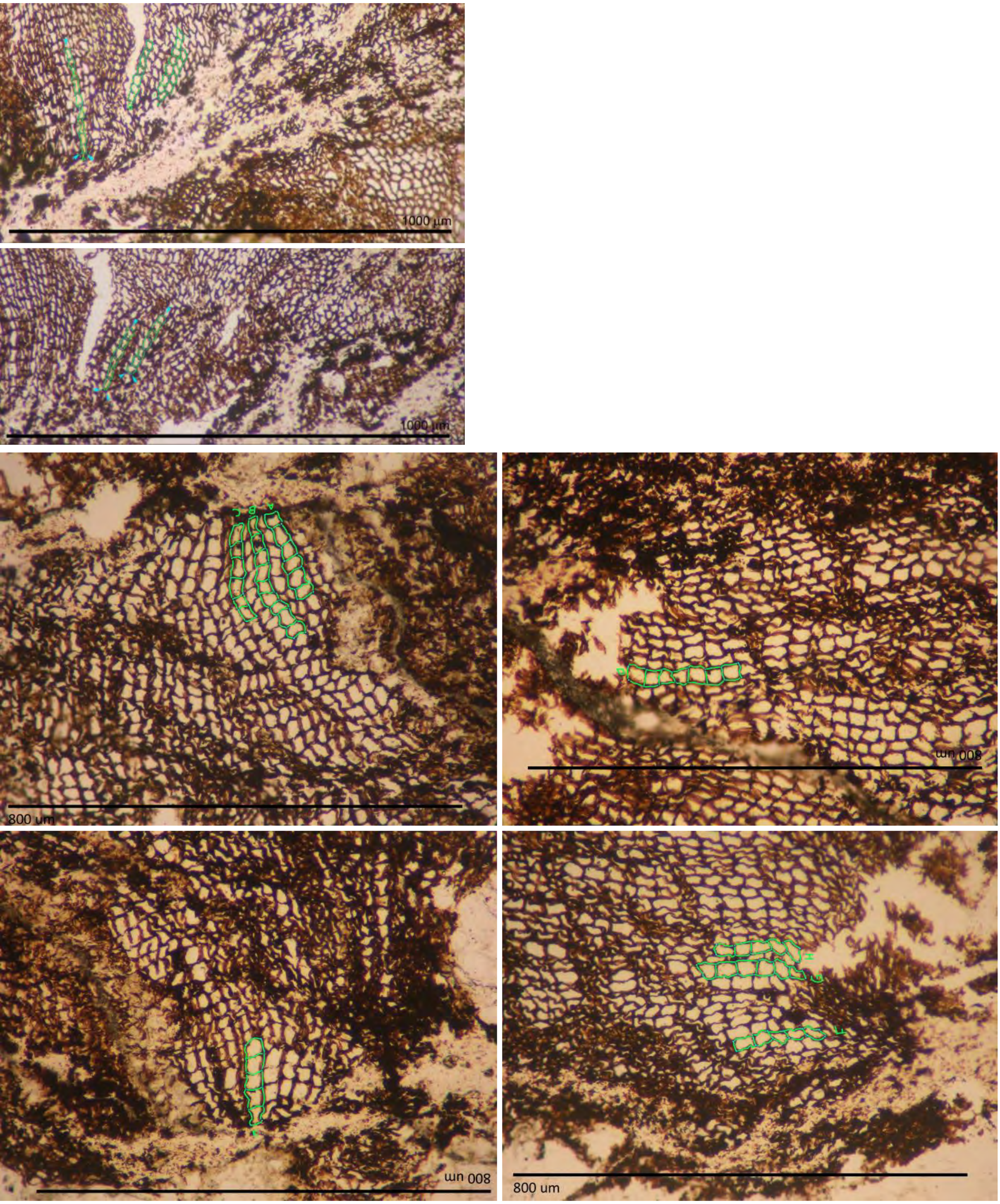

Specimen 6
557840 Gtop b
