# Supplementary material for: Characterizing and distinguishing the earliest woody euphyllophytes based on secondary xylem anatomy: method development and application
Source: Ann Bot. 2025 Jun 13;137(6):1602–23. doi: 10.1093/aob/mcaf122 (PMC13274980; doi:10.1093/aob/mcaf122)
Supplement: mcaf122_Supplementary_Data [file mcaf122_supplementary_data.zip › CasselmanTomescu2024_SupplementaryFigures_R1.docx]

SUPPLEMENTARY FIGURES

**Characterizing and distinguishing the earliest woody euphyllophytes based on secondary xylem anatomy: method development and application**

[Emma Casselman](mailto:etc107@humboldt.edu) and Alexandru M.F. Tomescu*

Department of Biological Sciences, California State Polytechnic University – Humboldt, Arcata, California 95521, U.S.A.

* Author for correspondence: [mihai@humboldt.edu](mailto:mihai@humboldt.edu)

**Supplementary Figure 1.** Notations for formula used to calculate corrected measurements for disrupted tracheids.

**Supplementary Figure 2.** Ratio of tangential tracheid size (T*_n_*) relative to that of the first tracheid in the file (T*_1_*) for extant genera.

**Supplementary Figure 3.** Ratio of tangential size between each tracheid (T*_n_*) and the one preceding it in the file (T*_n-1_*) for extant genera.

**Supplementary Figure 4.** Ratio of tangential tracheid size (T*_n_*) relative to that of its radial size (R*_n_*) for extant genera.

**Supplementary Figure 5.** Ratio of relative increase in tangential tracheid size (RIT*_n_*) relative to the radial size of the tracheid (R*_n_*) for extant genera.

**Supplementary Figure 6.** Ratio of the relative increase in tangential tracheid size RIT*_n_* compared to that of the previous tracheid in the file (RIT*_n-1_*) for extant genera.

**Supplementary Figure 7.** Effect of the sampling of different tracheid sequences within a file in extant taxa, for the ratio of tangential size between each tracheid (T*_n_*) and the one preceding it in the file (T*_n-1_*) and for tangential tracheid size (T*_n_*) against cumulative R.

**Supplementary Figure 8.** Effect of the sampling of additive sets of measurements along tracheid files in extant taxa, for the ratio of tangential size between each tracheid (T*_n_*) and the one preceding it in the file (T*_n-1_*) and for tangential tracheid size (T*_n_*) against cumulative R.

**Supplementary Figure 9.** Comparisons of new woody specimens from the Battery Point Formation based on multiple metrics.

**
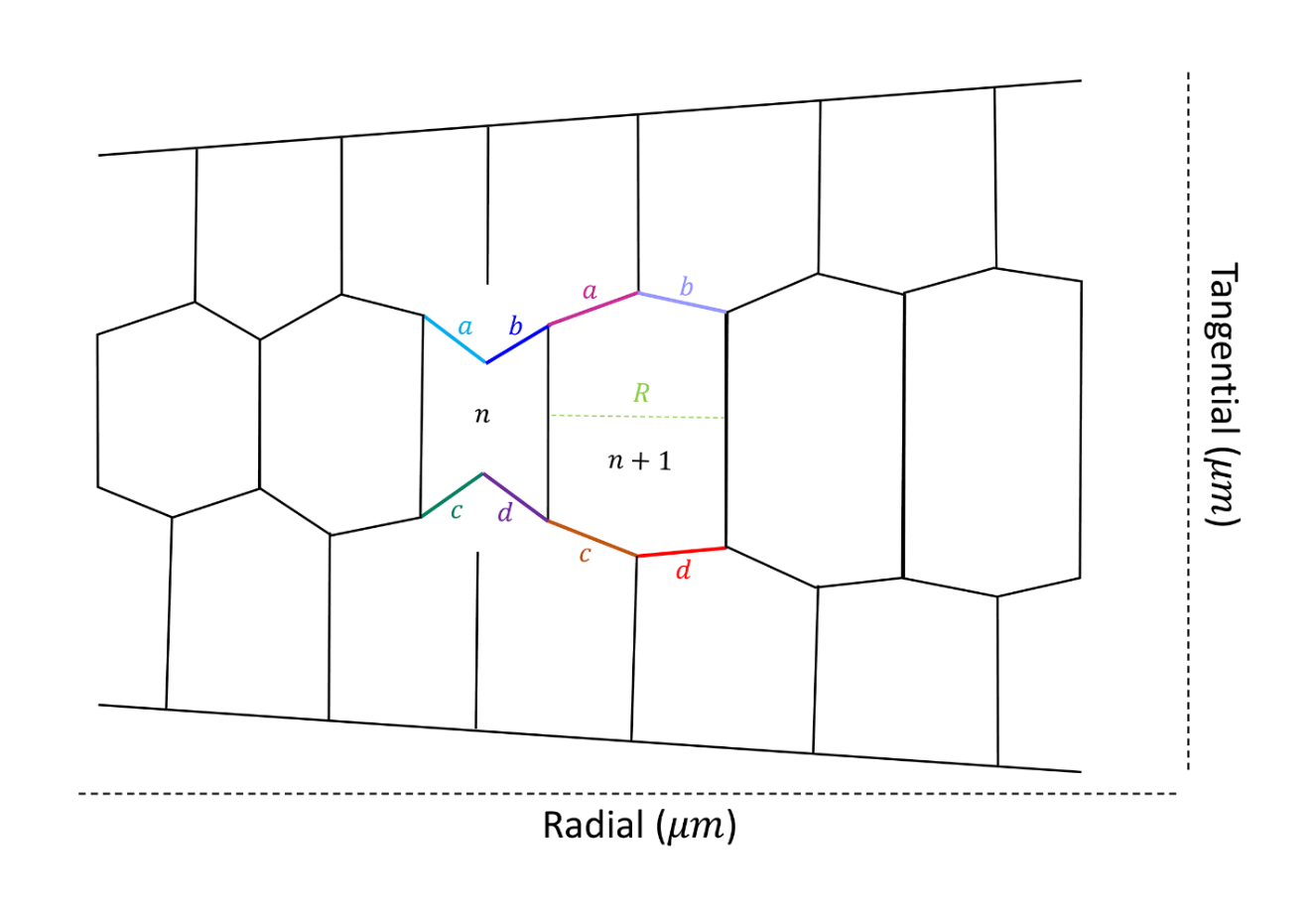
**

**Supplementary Figure 1.** Notations for formula used to calculate corrected measurements for disrupted tracheids based on comparisons and proportionality with adjacent tracheids. See Material and Methods section in the main text for detailed explanations.

**
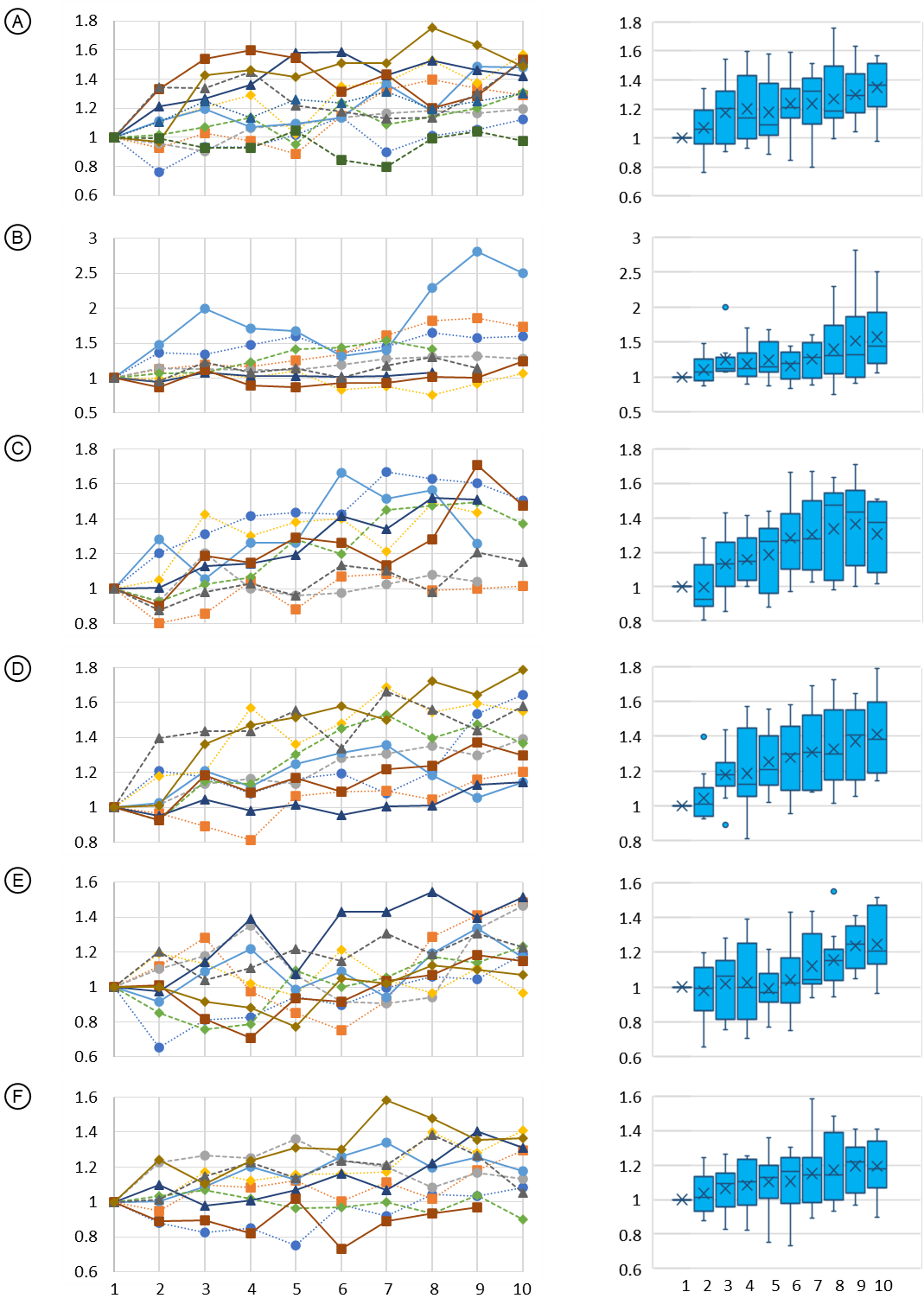
**

**Supplementary Figure 2.** Extant taxa. The ratio of tangential tracheid size (T*_n_*) relative to that of the first tracheid in the file (T*_1_*). (**A**) *Pinus* stem. (**B**) *Pinus* root. (**C**) *Sequoia* stem. (**D**) *Ginkgo* stem. (**E**) *Ginkgo* root. (**F**) *Ephedra* stem. Numbers on the x axis are tracheid ranks, from closest to the primary xylem = rank 1. Left panel – individual tracheid values in each file (each color, shape, and line combination represent a different tracheid file). Right panel – values of tracheids of same rank pooled for all tracheid files (one box plot for each tracheid rank); boxes include values between the first and third quartiles of the distribution (median excluded), whiskers represent maximum and minimum values in the distribution (exclusive of outliers), horizontal lines mark the medians, x’s mark the means, dots represent outliers.

**
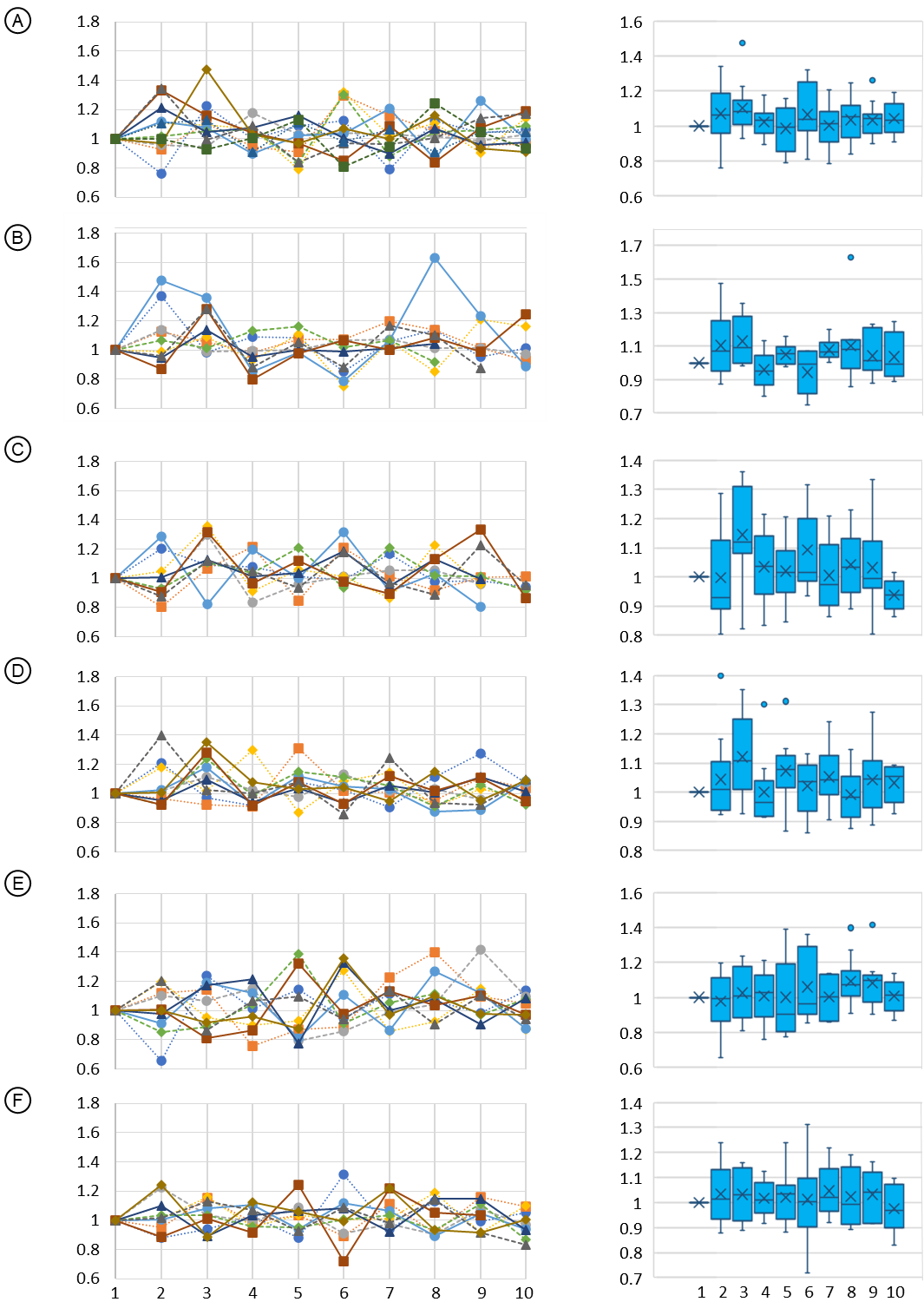
**

**Supplementary Figure 3.** Extant taxa. The ratio of tangential size between each tracheid (T*_n_*) and the one preceding it in the file (T*_n-1_*). (**A**) *Pinus* stem. (**B**) *Pinus* root. (**C**) *Sequoia* stem. (**D**) *Ginkgo* stem. (**E**) *Ginkgo* root. (**F**) *Ephedra* stem. Numbers on the x axis are tracheid ranks, from closest to the primary xylem = rank 1. Left panel – individual tracheid values in each file (each color, shape, and line combination represent a different tracheid file). Right panel – values of tracheids of same rank pooled for all tracheid files (one box plot for each tracheid rank); boxes include values between the first and third quartiles of the distribution (median excluded), whiskers represent maximum and minimum values in the distribution (exclusive of outliers), horizontal lines mark the medians, x’s mark the means, dots represent outliers.


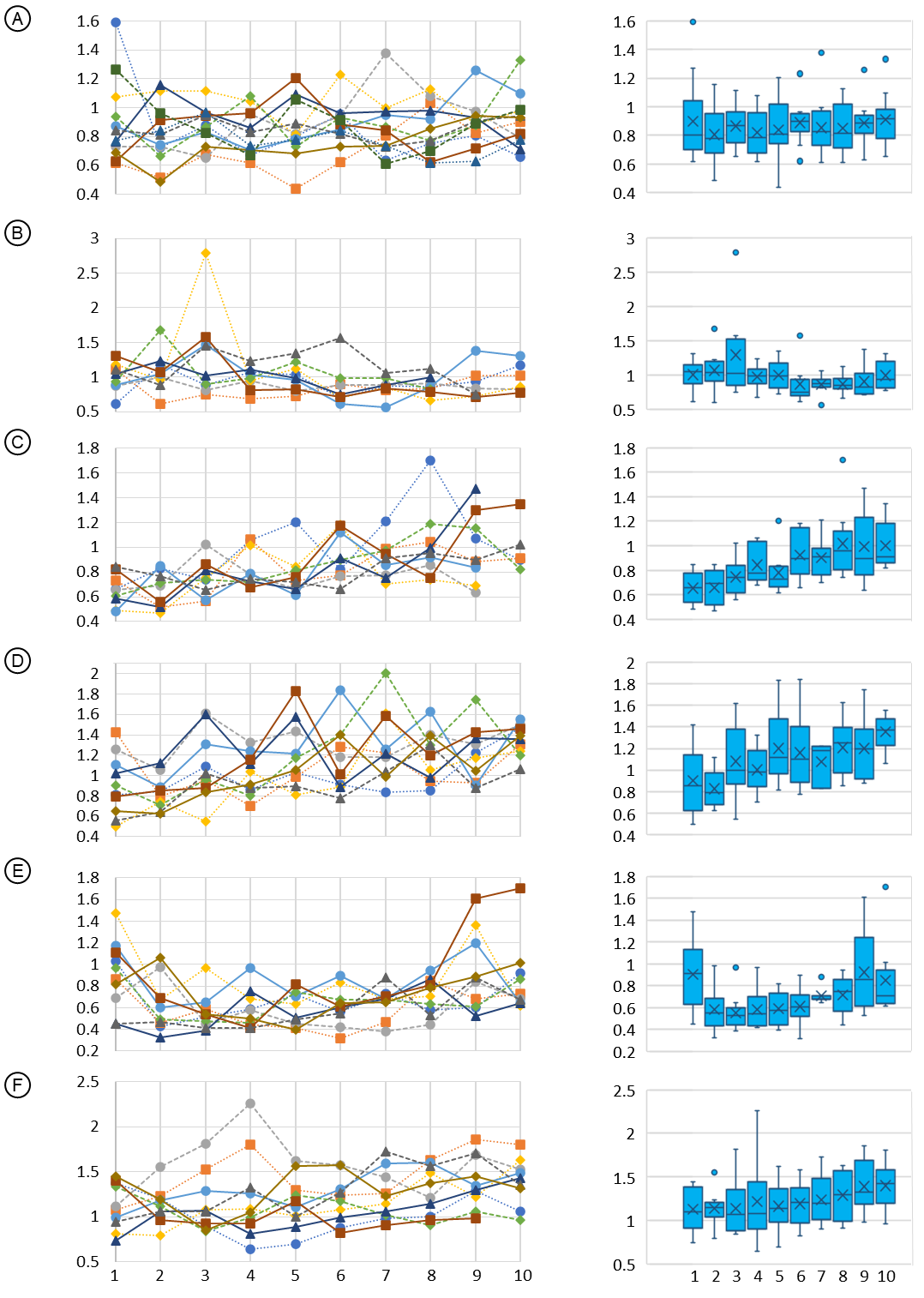


**Supplementary Figure 4.** Extant taxa. The ratio of tangential tracheid size (T*_n_*) relative to that of its radial size (R*_n_*). (**A**) *Pinus* stem. (**B**) *Pinus* root. (**C**) *Sequoia* stem. (**D**) *Ginkgo* stem. (**E**) *Ginkgo* root. (**F**) *Ephedra* stem. Numbers on the x axis are tracheid ranks, from closest to the primary xylem = rank 1. Left panel – individual tracheid values in each file (each color, shape, and line combination represent a different tracheid file). Right panel – values of tracheids of same rank pooled for all tracheid files (one box plot for each tracheid rank); boxes include values between the first and third quartiles of the distribution (median excluded), whiskers represent maximum and minimum values in the distribution (exclusive of outliers), horizontal lines mark the medians, x’s mark the means, dots represent outliers.

**
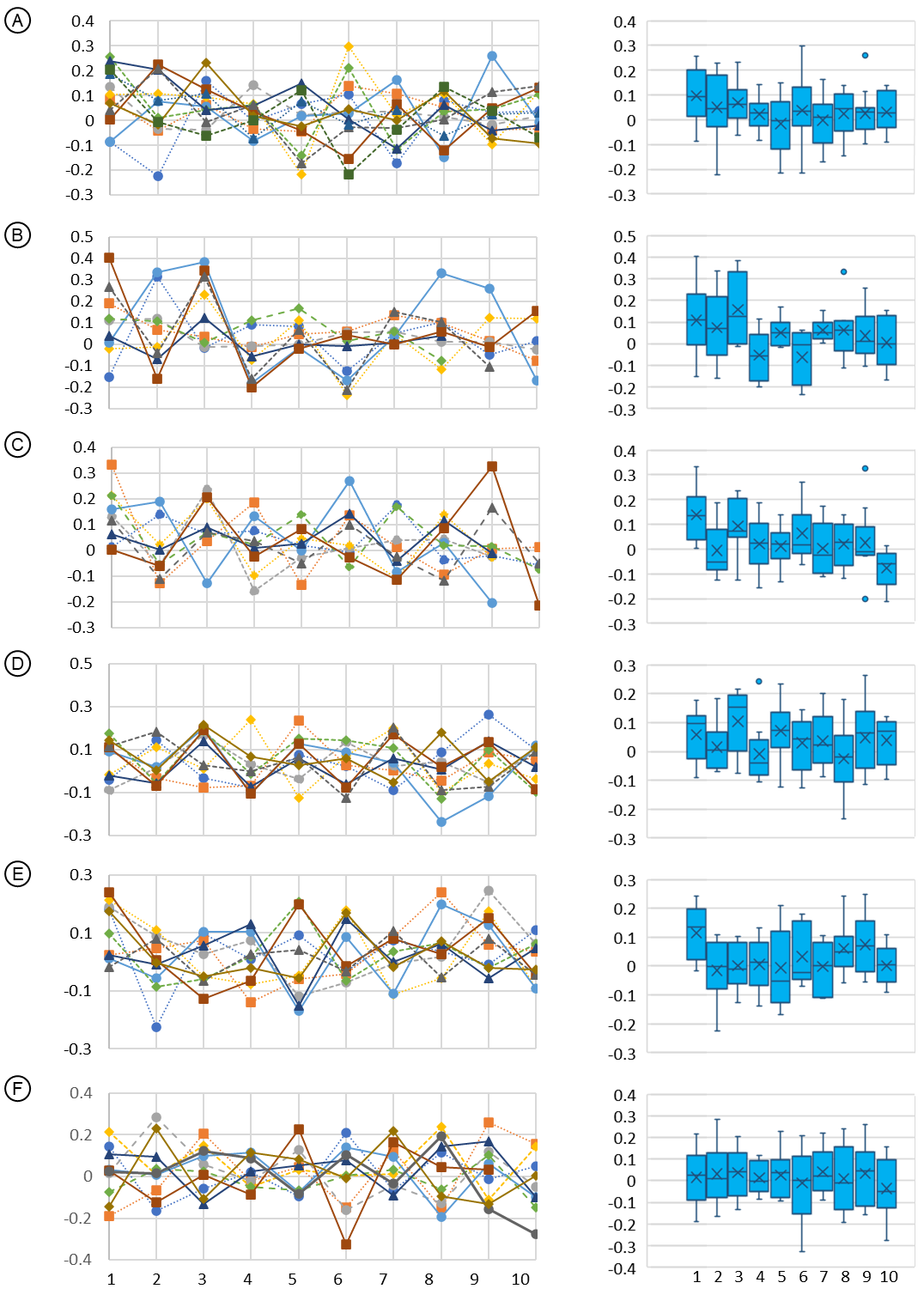
**

**Supplementary Figure 5.** Extant taxa. The ratio of relative increase in tangential tracheid size (RIT*_n_*) relative to the radial size of the tracheid (R*_n_*). (**A**) *Pinus* stem. (**B**) *Pinus* root. (**C**) *Sequoia* stem. (**D**) *Ginkgo* stem. (**E**) *Ginkgo* root. (**F**) *Ephedra* stem. Numbers on the x axis are tracheid ranks, from closest to the primary xylem = rank 1. Left panel – individual tracheid values in each file (each color, shape, and line combination represent a different tracheid file). Right panel – values of tracheids of same rank pooled for all tracheid files (one box plot for each tracheid rank); boxes include values between the first and third quartiles of the distribution (median excluded), whiskers represent maximum and minimum values in the distribution (exclusive of outliers), horizontal lines mark the medians, x’s mark the means, dots represent outliers.

**
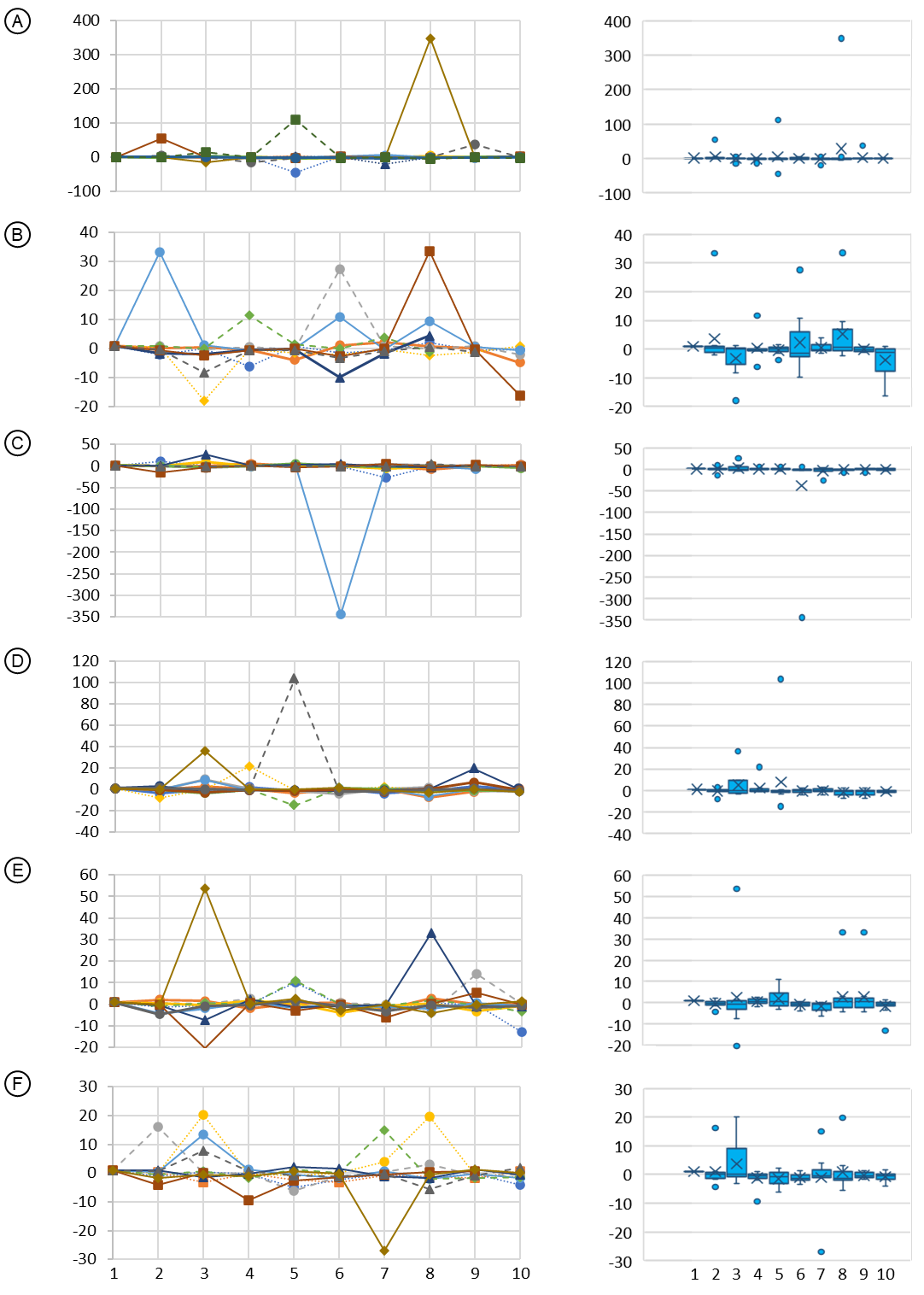
**

**Supplementary Figure 6.** Extant taxa. The ratio of the relative increase in tangential tracheid size RIT*_n_* compared to that of the previous tracheid in the file (RIT*_n-1_*). (**A**) *Pinus* stem. (**B**) *Pinus* root. (**C**) *Sequoia* stem. (**D**) *Ginkgo* stem. (**E**) *Ginkgo* root. (**F**) *Ephedra* stem. Numbers on the x axis are tracheid ranks, from closest to the primary xylem = rank 1. Left panel – individual tracheid values in each file (each color, shape, and line combination represent a different tracheid file). Right panel – values of tracheids of same rank pooled for all tracheid files (one box plot for each tracheid rank); boxes include values between the first and third quartiles of the distribution (median excluded), whiskers represent maximum and minimum values in the distribution (exclusive of outliers), horizontal lines mark the medians, x’s mark the means, dots represent outliers.

**
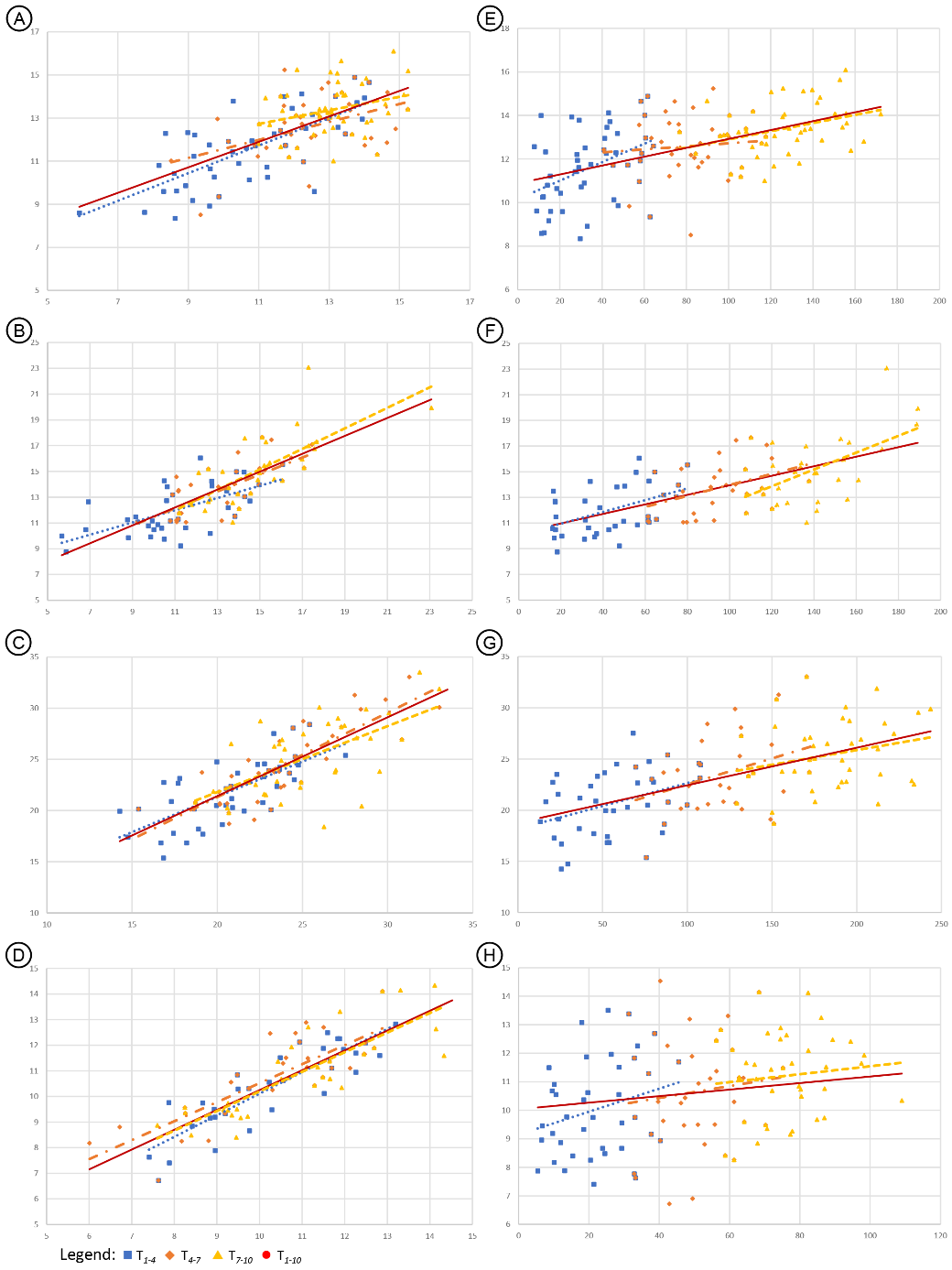
**

**Supplementary Figure 7.** Effect of the sampling of different tracheid sequences within a file. Successive sets of measurements along tracheid files (T*_1-4_* = blue, T*_4-7_* = orange, T*_7-10_* = yellow) treated as independent samples and compared to T*_1-10_* (red). (**A**-**D**) The ratio of tangential size between each tracheid (T*_n_*) and the one preceding it in the file (T*_n-1_*); (**E**-**H**) Tangential tracheid size (T*_n_*) against cumulative R. (**A**, **E**) *Pinus* stem; (**B**, **F**) *Sequoia* stem; (**C**, **G**) *Ginkgo* stem; (**D**, **H**) *Ephedra* stem. All values in µm.

**
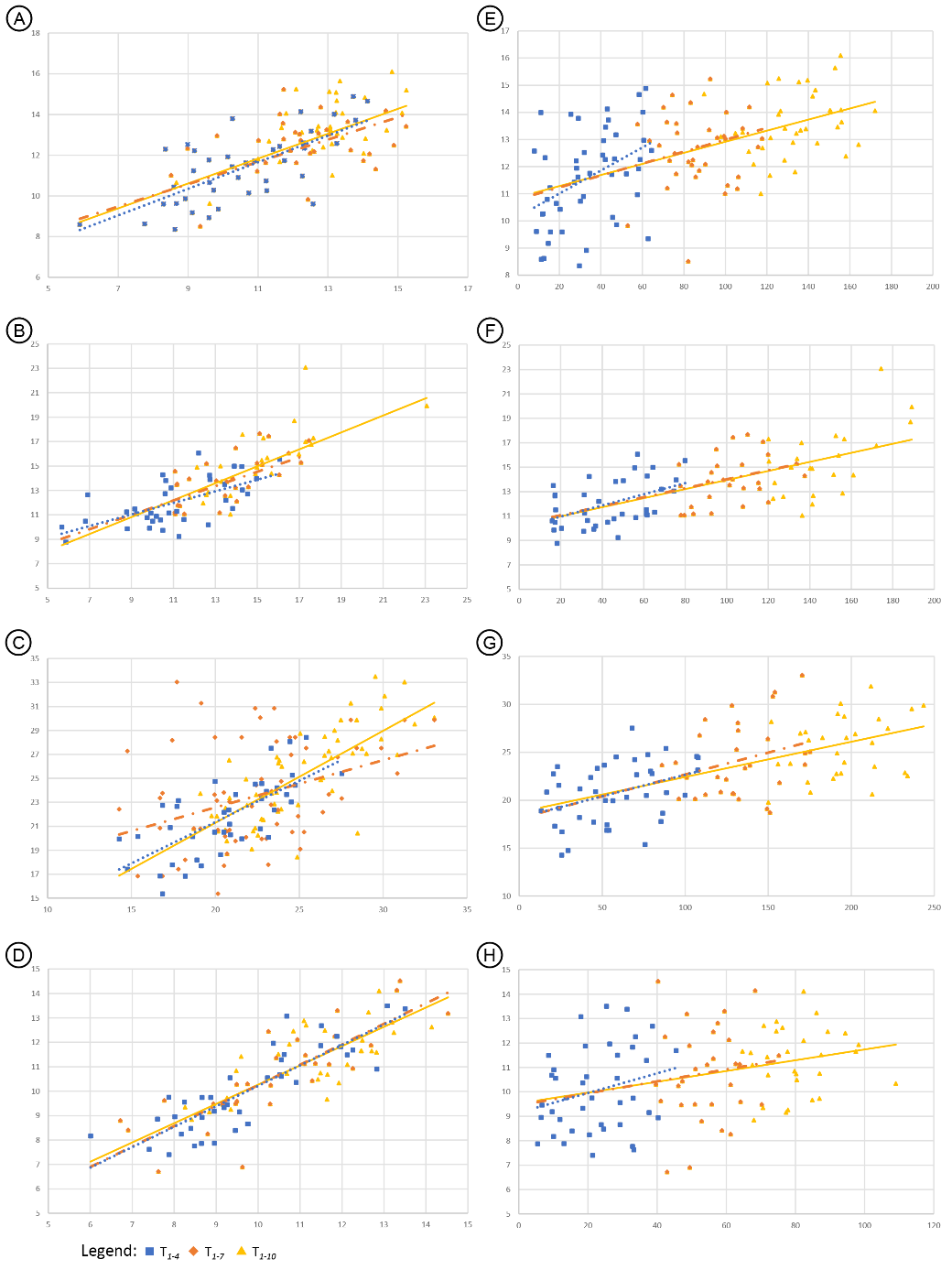
**

**Supplementary Figure 8.** Effect of the sampling of additive sets of measurements along tracheid files (i.e., T*_1-4_* , T*_1-7_* , T*_1-10_*). Additive sets of measurements along tracheid files (T*_1-4_* = blue, T*_1-7_* = orange, T*_1-10_* = yellow) treated as independent samples. (**A**-**D**) The ratio of tangential size between each tracheid (T*_n_*) and the one preceding it in the file (T*_n-1_*); (**E**-**H**) Tangential tracheid size (T*_n_*) against cumulative R. (**A**, **E**) *Pinus* stem; (**B**, **F**) *Sequoia* stem; (**C**, **G**) *Ginkgo* stem; (**D**, **H**) *Ephedra* stem. All values in µm.

**
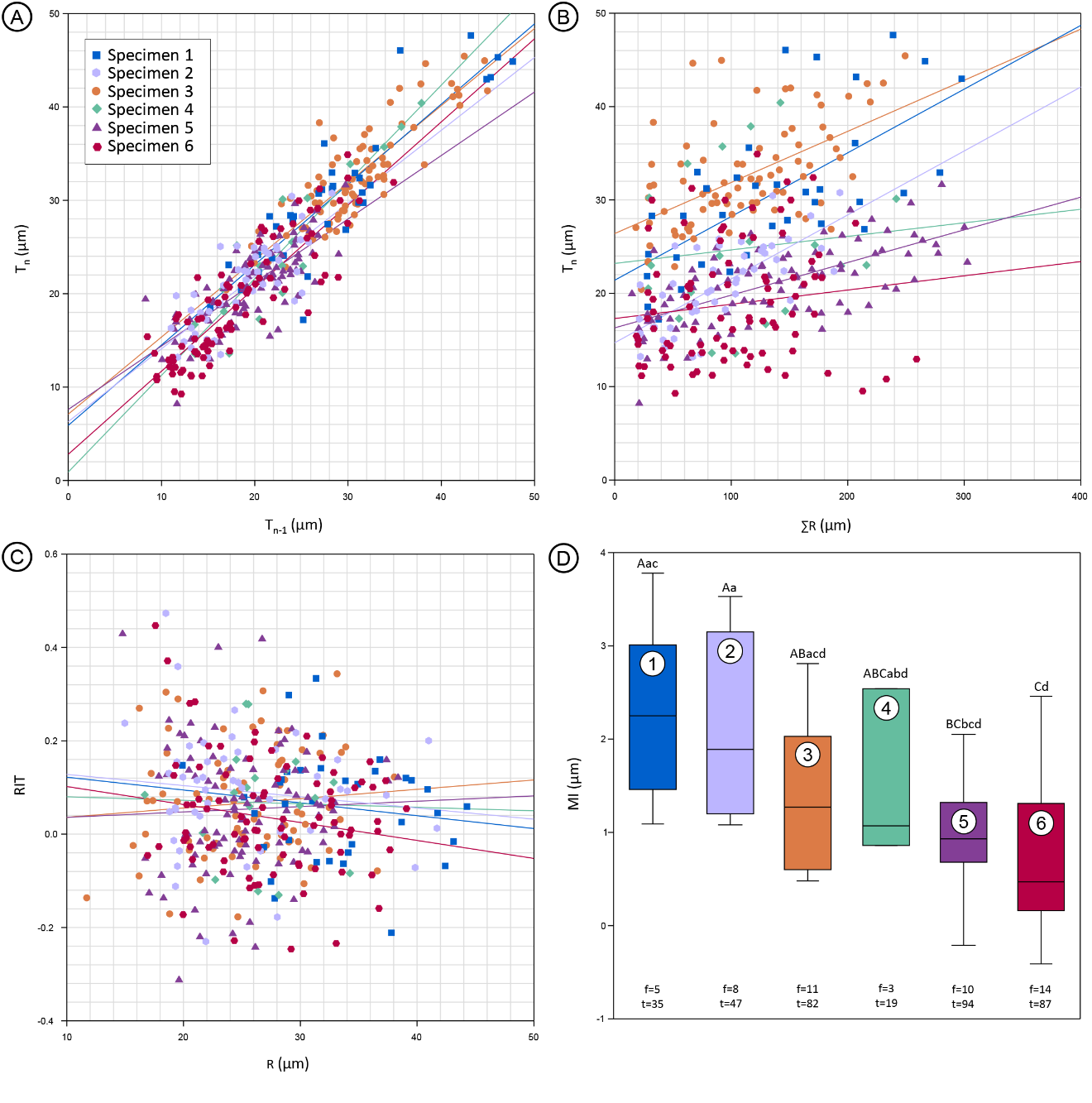
**

**Supplementary Figure 9.** Comparisons of new woody specimens from the Battery Point Formation. (**A**) The ratio of tangential size between each tracheid (T*_n_*) and the one preceding it (T*_n-1_*). (**B**) Tangential tracheid size (T*_n_*) against cumulative R. (**C**) The increase in tangential tracheid size (RIT*_n_*) relative to the radial size (R*_n_*). (**D**) Mean increase in tangential tracheid size along radial files (MI). Letters indicate statistically significant differences between taxa (reject H_0_ at α = 0.05): uppercase letters = Aspin-Welch unequal-variance t-test; lowercase letters = Kolmogorov-Smirnov test for distributions.
