# Supplementary material for: Characterizing and distinguishing the earliest woody euphyllophytes based on secondary xylem anatomy: method development and application
Source: Ann Bot. 2025 Jun 13;137(6):1602–23. doi: 10.1093/aob/mcaf122 (PMC13274980; doi:10.1093/aob/mcaf122)
Supplement: mcaf122_Supplementary_Data [file mcaf122_supplementary_data.zip › CasselmanTomescu2024_SupplementaryNote.docx]

SUPPLEMENTARY NOTE

**Characterizing and distinguishing the earliest woody euphyllophytes based on secondary xylem anatomy: method development and applications**

[Emma Casselman](mailto:etc107@humboldt.edu) and Alexandru M.F. Tomescu*

Department of Biological Sciences, California State Polytechnic University – Humboldt, Arcata, California 95521, U.S.A.

* Author for correspondence: [mihai@humboldt.edu](mailto:mihai@humboldt.edu)

**Regression equations for all taxa and metrics.**

**1. Extant taxa**

*T_n_ vs T_n-1_*

*Pinus* stem Y = 0.7630x + 3.4413 R² = 0.4657

*Pinus* root Y = 0.9948x + 1.4678 R² = 0.9078

*Sequoia* stem Y = 0.6941x + 4.5650 R² = 0.6024

*Ginkgo* stem Y = 0.7695x + 6.0323 R² = 0.6555

*Ginkgo* root Y = 0.2765x + 18.655 R² = 0.1182

*Ephedra* stem Y = 0.7739x + 2.5066 R² = 0.6733

*T_n_ vs Cumulative R*

*Pinus* stem Y = 0.0204x + 10.874 R² = 0.3023

*Sequoia* stem Y = 0.0371x + 10.227 R² = 0.4446

*Ginkgo* stem Y = 0.026x + 19.818 R² = 0.3555

*Ephedra* stem Y = 0.0207x + 9.5768 R² = 0.1109

*RIT_n_ vs R*

*Pinus* stem Y = -0.003x + 0.0797 R² = 0.0053

*Pinus* root Y = -0.0072x + 0.197 R² = 0.0590

*Sequoia* stem Y = 0.0041x - 0.0313 R² = 0.0115

*Ginkgo* stem Y = -0.002x + 0.0701 R² = 0.0041

*Ginkgo* root Y = -0.0035x + 0.166 R² = 0.1047

*Ephedra* stem Y = 0.014x - 0.1127 R² = 0.0274

*RIT_n_ vs T_n_*

*Pinus* stem Y = 0.0167x - 0.1782 R² = 0.0948

*Pinus* root Y = 0.0096x - 0.1432 R² = 0.0517

*Sequoia* stem Y = 0.0073x - 0.063 R² = 0.0278

*Ginkgo* stem Y = 0.0057x - 0.1087 R² = 0.0371

*Ginkgo* root Y = 0.0075x - 0.1721 R² = 0.1413

*Ephedra* stem Y = 0.0164x - 0.164 R² = 0.0494

Successive sets of measurements along tracheid files (i.e., T*_1-4_* , T*_4-7_* , T*_7-10_*) treated as independent samples and compared to T*_1-10_*

*Pinus* stem *T_n_ vs T_n-1_*

T*_1-4_* Y = 0.64x + 4.6813 R² = 0.5257

T*_4-7_* Y = 0.4159x + 7.4063 R² = 0.1935

T*_7-10_* Y = 0.314x + 9.2736 R² = 0.0869

T*_1-10_* Y = 0.5907x + 5.3967 R² = 0.4430

*Sequoia* stem *T_n_ vs T_n-1_*

T*_1-4_* Y = 0.4741x + 6.7673 R² = 0.3964

T*_4-7_* Y = 0.7944x + 3.2456 R² = 0.5138

T*_7-10_* Y = 0.6941x + 4.565 R² = 0.6024

T*_1-10_* Y = 0.5907x + 5.3967 R² = 0.4430

*Ginkgo* stem *T_n_ vs T_n-1_*

T*_1-4_* Y = 0.6889x + 7.5636 R² = 0.4930

T*_4-7_* Y = 0.8252x + 4.8064 R² = 0.6935

T*_7-10_* Y = 0.6444x + 8.9079 R² = 0.3674

T*_1-10_* Y = 0.7695x + 6.0323 R² = 0.6555

*Ephedra* stem *T_n_ vs T_n-1_*

T*_1-4_* Y = 0.8426x + 1.6769 R² = 0.7521

T*_4-7_* Y = 0.7425x + 3.091 R² = 0.6728

T*_7-10_* Y = 0.7657x + 2.5353 R² = 0.6977

T*_1-10_* Y = 0.7739x + 2.5066 R² = 0.6733

*Pinus* stem *T_n_ vs Cumulative R*

T*_1-4_* Y = 0.0423x + 10.161 R² = 0.1823

T*_4-7_* Y = 0.007x + 12.026 R² = 0.0095

T*_7-10_* Y = 0.0187x + 11.025 R² = 0.1258

T*_1-10_* Y = 0.0204x + 10.874 R² = 0.3023

*Sequoia* stem *T_n_ vs Cumulative R*

T*_1-4_* Y = 0.0459x + 10.028 R² = 0.2245

T*_4-7_* Y = 0.0428x + 9.6899 R² = 0.2074

T*_7-10_* Y = 0.0656x + 5.9905 R² = 0.3170

T*_1-10_* Y = 0.6941x + 4.565 R² = 0.6024

*Ginkgo* stem *T_n_ vs Cumulative R*

T*_1-4_* Y = 0.0449x + 18.16 R² = 0.1491

T*_4-7_* Y = 0.0504x + 17.486 R² = 0.1451

T*_7-10_* Y = 0.0284x + 20.184 R² = 0.0605

T*_1-10_* Y = 0.0368x + 18.752 R² = 0.3423

*Ephedra* stem *T_n_ vs Cumulative R*

T*_1-4_* Y = 0.0404x + 9.145 R² = 0.0648

T*_4-7_* Y = 0.0209x + 9.5949 R² = 0.0154

T*_7-10_* Y = 0.0209x + 9.5949 R² = 0.0154

T*_1-10_* Y = 0.0114x + 10.041 R² = 0.0300

Additive sets of measurements along tracheid files (i.e., T*_1-4_* , T*_1-7_* , T*_1-10_*)

*Pinus* stem *T_n_ vs T_n-1_*

T*_1-4_* Y = 0.6538x + 4.4644 R² = 0.4941

T*_1-7_* Y = 0.5484x + 5.6371 R² = 0.4132

T*_1-10_* Y = 0.6118x + 5.0998 R² = 0.4537

*Sequoia* stem *T_n_ vs T_n-1_*

T*_1-4_* Y = 0.4741x + 6.7673 R² = 0.3964

T*_1-7_* Y = 0.5897x + 5.6886 R² = 0.5152

T*_1-10_* Y = 0.4741x + 6.7673 R² = 0.3964

*Ginkgo* stem *T_n_ vs T_n-1_*

T*_1-4_* Y = 0.6889x + 7.5636 R² = 0.4930

T*_1-7_* Y = 0.395x + 14.672 R² = 0.1379

T*_1-10_* Y = 0.7696x + 5.8844 R² = 0.6274

*Ephedra* stem *T_n_ vs T_n-1_*

T*_1-4_* Y = 0.838x + 1.8371 R² = 0.7109

T*_1-7_* Y = 0.837x + 1.8737 R² = 0.7121

T*_1-10_* Y = 0.7899x + 2.368 R² = 0.6791

*Pinus* stem *T_n_ vs Cumulative R*

T*_1-4_* Y = 0.0423x + 10.161 R² = 0.1823

T*_1-7_* Y = 0.022x + 10.797 R² = 0.1711

T*_1-10_* Y = 0.0423x + 10.161 R² = 0.1823

*Sequoia* stem *T_n_ vs Cumulative R*

T*_1-4_* Y = 0.0459x + 10.028 R² = 0.2245

T*_1-7_* Y = 0.0368x + 10.33 R² = 0.3445

T*_1-10_* Y = 0.0371x + 10.227 R² = 0.4446

*Ginkgo* stem *T_n_ vs Cumulative R*

T*_1-4_* Y = 0.0449x + 18.16 R² = 0.1491

T*_1-7_* Y = 0.0457x + 18.12 R² = 0.2898

T*_1-10_* Y = 0.0368x + 18.752 R² = 0.3423

*Ephedra* stem *T_n_ vs Cumulative R*

T*_1-4_* Y = 0.0404x + 9.145 R² = 0.0648

T*_1-7_* Y = 0.0245x + 9.4491 R² = 0.0641

T*_1-10_* Y = 0.0221x + 9.5269 R² = 0.1111

**2. Previously described fossil taxa**

*T_n_ vs T_n-1_*

*Psilophyton* Y = 0.7465x + 15.17 R² = 0.5616

*Armoricaphyton* Y = 0.9449x + 6.2448 R² = 0.9320

*Franhueberia* Y = 0.6901x + 7.4011 R² = 0.5546

*Gmujij* Y = 0.683x + 6.7328 R² = 0.4858

*T_n_ vs Cumulative R*

*Psilophyton* Y = 0.0855x + 31.407 R² = 0.3277

*Armoricaphyton* Y = 0.0758x + 26.655 R² = 0.6900

*Franhueberia* Y = 0.0243x + 17.49 R² = 0.4146

*Gmujij* Y = 0.0068x + 19.822 R² = 0.0140

*RIT_n_ vs T_n_*

*Psilophyton* Y = -0.0009x + 0.1449 R² = 0.0025

*Armoricaphyton* Y = -0.0016x + 0.1612 R² = 0.0316

*Franhueberi* Y = -0.0046x + 0.1676 R² = 0.0378

*Gmujij* Y = 0.0016x - 0.0047 R² = 0.0051

**3. New fossil specimens**

*T_n_ vs T_n-1_*

Specimen 1 Y = 0.8592x + 5.9153 R² = 0.8025

Specimen 2 Y = 0.7791x + 6.3258 R² = 0.6089

Specimen 3 Y = 0.8264x + 7.0908 R² = 0.7401

Specimen 4 Y = 1.0355x + 0.9337 R² = 0.8448

Specimen 5 Y = 0.6801x + 7.6384 R² = 0.5691

Specimen 6 Y = 0.8892x + 2.8415 R² = 0.7622

*T_n_ vs Cumulative R*

Specimen 1 Y = 0.0684x + 21.3821 R² = 0.4489

Specimen 2 Y = 0.0686x + 14.6851 R² = 0.5241

Specimen 3 Y = 0.0548x + 26.3787 R² = 0.3195

Specimen 4 Y = 0.0144x + 23.2259 R² = 0.0126

Specimen 5 Y = 0.0349x + 16.3141 R² = 0.4213

Specimen 6 Y = 0.0151x + 17.3436 R² = 0.0170

*RIT_n_ vs T_n_*

Specimen 1 Y = -0.0027x + 0.1483 R² = 0.0186

Specimen 2 Y = -0.0024x + 0.1526 R² = 0.0155

Specimen 3 Y = 0.0020x + 0.0154 R² = 0.0087

Specimen 4 Y = -0.0007x + 0.0867 R² = 0.0007

Specimen 5 Y = 0.0012x + 0.0234 R² = 0.0018

Specimen 6 Y = -0.0039x + 0.1410 R² = 0.0317
